# A novel role for Friend of GATA1 (FOG-1) in regulating cholesterol transport in murine erythropoiesis

**DOI:** 10.1371/journal.pgen.1011617

**Published:** 2025-03-06

**Authors:** Ioannis-Marios Roussis, David J. Pearton, Umar Niazi, Grigorios Tsaknakis, Giorgio L. Papadopoulos, Riley Cook, Mansoor Saqi, Jiannis Ragoussis, John Strouboulis

**Affiliations:** 1 Red Cell Haematology Lab, Comprehensive Cancer Centre, School of Cancer and Pharmaceutical Sciences, Faculty of Life Sciences and Medicine, King’s College London, London, United Kingdom; 2 Department of Biology, University of Crete, Heraklion, Crete, Greece; 3 Translational Bioinformatics, National Institute for Health Research Biomedical Centre, Guy’s and St Thomas’ NHS Foundation Trust and King’s College London, London, United Kingdom; 4 Institute of Molecular Biology and Biotechnology, Foundation for Research & Technology Hellas, Heraklion, Crete, Greece; 5 Bone Marrow Failure Group, Comprehensive Cancer Centre, School of Cancer and Pharmaceutical Sciences, Faculty of Life Sciences and Medicine, King’s College London, London, United Kingdom; 6 Department of Human Genetics, McGill University and McGill Genome Centre, Montreal, Quebec, Canada; Indian Institute of Science Education and Research Mohali, INDIA

## Abstract

Friend of GATA1 (FOG-1) is an essential transcriptional co-factor of the master erythroid transcription factor GATA1. The knockout of the *Zfpm1* gene, coding for FOG-1, results in early embryonic lethality due to anemia in mice, similar to the embryonic lethal phenotype of the *Gata1* gene knockout. However, a detailed molecular analysis of the *Zfpm1* knockout phenotype in erythropoiesis is presently incomplete. To this end, we used CRISPR/Cas9 to knockout *Zfpm1* in mouse erythroleukemic (MEL) cells. Phenotypic characterization of DMSO-induced terminal erythroid differentiation showed that the *Zfpm1* knockout MEL cells did not progress past the proerythroblast stage of differentiation. Expression profiling of the *Zfpm1* knockout MEL cells by RNAseq showed a lack of up-regulation of erythroid-related gene expression profiles. Bioinformatic analysis highlighted cholesterol transport as a pathway affected in the *Zfpm1* knockout cells. Moreover, we show that the cholesterol transporters *Abca1* and *Ldlr* fail to be repressed during erythroid differentiation in *Zfpm1* knockout cells, resulting in higher intracellular lipid levels and higher membrane fluidity. We also show that in FOG-1 knockout cells, the nuclear levels of SREBP2, a key transcriptional regulator of cholesterol biosynthesis and transport, are markedly increased. On the basis of these findings we propose that FOG-1 (and, potentially, GATA1) regulate cholesterol homeostasis during erythroid differentiation directly through the down regulation of cholesterol transport genes and indirectly, through the repression of the SREBP2 transcriptional activator of cholesterol homeostasis. Taken together, our work provides a molecular basis for understanding FOG-1 functions in erythropoiesis and reveals a novel role for FOG-1 in cholesterol transport.

## Author summary

Erythropoiesis is the process by which the oxygen-carrying red blood cells (RBCs) are produced in our body. Friend of GATA1 (FOG-1) and its partner GATA1, are key molecules that control erythropoiesis by coordinating the activity of the many genes that are required for the production of functional RBCs. To obtain more insight as to how FOG-1 works in erythropoiesis, we knocked out the FOG-1 gene in mouse immature red cells and asked the question of how this affects RBC production in a Petri dish. As expected, we found that the FOG-1 knockout cells cannot progress to produce mature red cells. By analyzing the differences in the RNA profiles between the normal and the FOG-1 knockout cells, we saw for the first time that proteins called cholesterol transporters were affected, in that they were not turned off, as would be the case in the normal cells. Cholesterol is a naturally occurring fat-like substance utilized in the cell membrane to enhance cell flexibility. This is a key RBC property that allows them to squeeze through tight spaces around the body. Our findings are important in understanding (i) key FOG-1 functions in RBC production and (ii) cholesterol functions in RBCs.

## Introduction

Friend of GATA1 (FOG-1) is the founding member of the FOG (Friend of GATA) family of proteins, an evolutionarily conserved family of transcriptional co-factors that bind to the N-terminal zinc finger domain of the critical GATA family of transcription factors, regulating their activity in various cell lineages and developmental pathways [1]. FOG-1 was first isolated in a yeast two-hybrid screen using the N-terminal zinc finger of the key erythro/megakaryocytic transcription factor GATA1 as bait [2]. Murine FOG-1 is a large protein (995 amino acids) encoded by the *Zfpm1* gene, and has an unusual structure in that it contains nine multi-type zinc fingers (Zfs), four resembling the classical C2H2 configuration (Zf2, Zf3, Zf4, Zf8) and five unusual C2HC variants (Zf1, Zf5, Zf6, Zf7, Zf9), in which the final zinc binding histidine is replaced by a cysteine [2]. FOG-1 is not known to bind directly to DNA but, instead, is thought to be recruited to DNA through its interaction with GATA1 [3]. FOG-1 also features two domains responsible for protein-protein interactions, namely, an N-terminal domain mediating interactions with the remodeling and histone deacetylase (NuRD) complex [4,5], and a second domain between Zf6 and Zf7 mediating interactions with the CtBP corepressor complex [6]. More recently, FOG-1 was also shown to contain a PR (PRDI-BF1 and RIZ homology) domain near its N-terminus, though its function remains unclear [7]. FOG-1 protein is expressed as two translational isoforms, due to a downstream internal initiation codon (ATG) within the canonical transcript [8]. The shorter isoform is an N-terminally truncated version of FOG-1 that retains all nine zinc fingers but lacks the NuRD-binding N-terminal domain, while maintaining its capacity to interact with GATA1 and the CtBP complex.

The spatio-temporal expression profile of FOG-1 is almost identical to that of GATA1 [2]. In both primitive (fetal) and definitive (adult) hematopoiesis, GATA1 and FOG-1 are expressed at high levels in the erythroid and megakaryocytic cell lineages. Furthermore, the overlapping functions of FOG-1 and GATA1 in the two hematopoietic lineages were revealed from the knockout phenotypes of the two genes in mice. Specifically, the targeted deletion of *Gata1* causes early embryonic lethality around embryonic days 10.5-11.5 from severe anemia, due to erythroid differentiation arresting at the proerythroblast stage [9,10]. GATA1’s role in megakaryopoiesis was also established in mice harboring a megakaryocyte-selective GATA1 knockout, which leads to megakaryocytic hyperproliferation and defective cytoplasmic maturation [11,12]. Similar to the *Gata1* knockout phenotype, deletion of the *Zfpm1* gene in mice resulted in embryonic lethality between embryonic days 10.5 to 12.5, due to severe anemia [3]. However, compared to the *Gata1* knockout, ablation of FOG-1 had a more profound effect on megakaryopoiesis [3].

It is now well-established that a physical interaction between FOG-1 and GATA1 is essential for physiological erythro/megakaryopoiesis. A yeast two-hybrid altered specificity mutant screen, identified specific amino acid residues in the N-terminal zinc finger domain of GATA1, especially valine at position 205, as being essential for interaction with FOG-1 and for erythro/megakaryopoiesis *in vitro* and *in vivo* [13,14]. Importantly, the inherited GATA1 V205M mutation is associated with familial dyserythropoietic anemia and thrombocytopenia in patients [15], once again highlighting the importance of a functional FOG-1/GATA1 interaction in hematopoiesis.

The transcriptional co-factor functions of FOG-1 with GATA1 in erythropoiesis have been studied extensively [1]. It is well established that FOG-1 and GATA1 serve as both activators and repressors of gene expression in erythropoiesis [4,13]. It has also been proposed that FOG-1 serves as a chromatin facilitator allowing GATA1 chromatin occupancy of target genes, in a context-dependent manner [16–18]. A FOG-1/GATA1 interaction has also been shown to be essential for DNA looping between distal enhancer and promoters in the murine β-globin and *Kit* gene loci [19,20]. It has also been shown that FOG-1 mediates interactions between the NuRD complex and GATA1 in repressing or activating gene expression [4,5,21]. FOG-1 has also been described to interact with the CtBP co-repressor and the TACC3 co-factor, however the functional significance of these interactions in erythropoiesis remains unclear [6,22].

Despite all this information regarding FOG-1 and in contrast to the GATA1 erythroid knockout phenotype which has been analyzed extensively in a number of erythroid models, less is known about the molecular basis of the FOG-1 knockout phenotype in erythropoiesis. Here, we used CRISPR/Cas9 mediated gene editing to knock out the *Zfpm1* gene in murine erythroleukemic (MEL) cells coupled with expression profiling by RNAseq, to provide molecular insight into the FOG-1 knockout phenotype in erythroid cells. This work revealed a previously unknown function of FOG-1 in regulating cholesterol transport in erythropoiesis.

## Results

### CRISPR/Cas9 generated FOG-1 knockout MEL cell lines

**Fig 1:**
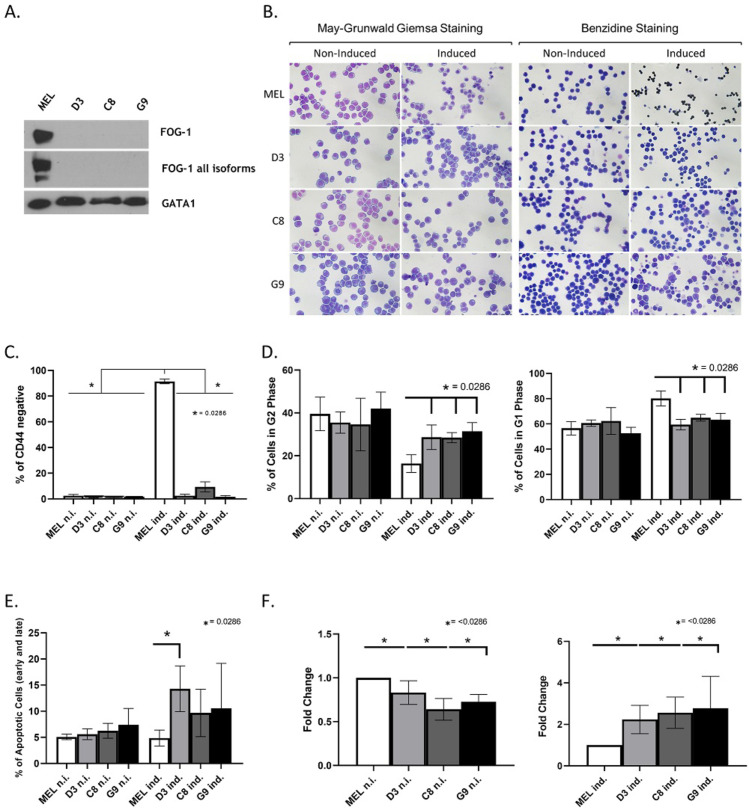
Phenotypic characterization of terminal erythroid differentiation in FOG-1 knockout MEL cell clones. **(A)** Western immunoblot analysis showing the lack of FOG-1 protein in the MEL knockout (KO) clones D3, C8 and G9, compared to wild type (WT) MEL cells. Two antibodies recognizing the FOG-1 long isoform (top panel) and all FOG-1 isoforms (middle panel) were used. GATA1 was used as a protein loading control. **(B)** May-Grunwald Giemsa staining (left panels) of non-induced and DMSO-induced WT MEL cells and FOG-1 KO clones D3, C8 and G9. Benzidine staining (right panels) shows a lack of hemoglobin accumulation in the induced FOG-1 KO MEL cells, in contrast to the dark brown stained hemoglobinized cells in the DMSO-induced WT MEL panel. **(C)** Quantitation of DMSO-induced MEL differentiation as a percentage of cells that are negative for the CD44 cell surface marker, which is extinguished with terminal differentiation. Data were obtained by flow cytometry analysis (n = 4). **(D)** Cell cycle analysis of differentiating WT and FOG-1 KO MEL cells using propidium iodide and flow cytometry (n = 4). Left panel: percentage of cells in G1 phase; right panel: percentage of cells in G2 phase. **(E)** Quantitation of apoptosis/necrosis as a percentage of total cells using annexin and propidium iodide measured by flow cytometry (n = 4). **(F)** Fold-change quantitation of Reactive Oxygen Species (ROS) in FOG-1 KO MEL cells compared to WT MEL, measured by flow cytometry in non-induced (left panel) and DMSO-induced (right panel) cells (n = 4). n.i.: non-induced cells; ind.: DMSO-induced cells.

We used CRISPR/Cas9 to knockout (KO) the *Zfpm1* gene in mouse erythroleukemic (MEL) cells, which serve as a cellular model for murine terminal erythropoiesis. Stably transfected MEL cells were G418 selected and single cell clones were isolated by sorting for GFP expression. The efficiency of knocking out *Zfpm1* was assessed by Western immunoblot analysis of FOG-1 expression. Using two different anti-FOG-1 antibodies, no FOG-1 protein was detected in three KO MEL clones tested (Fig 1A). We proceeded to characterize in greater detail the edits in the *Zfpm1* gene in the three FOG-1 KO clones by PCR exon analysis of the first five 5’ exons using genomic DNA (S1A Fig). From this analysis it is evident that exons 2 and 3 can be used to distinguish between the three clones, since exon 2 is deleted only in clone D3 and exon 3 is intact only in clone G9 (S1C Fig). Furthermore, the alignment of all *Zfpm1* sequences extracted from the RNAseq analysis of the three FOG-1 KO clones (see below), is in agreement with the PCR exon analysis (S1B Fig; example shown for exon 4). Lastly, by utilizing these sequences to check for open reading frames (ORFs), it was evident that in all three clones the FOG-1 protein is completely knocked out and there is no expression of a truncated version of FOG-1, as a stop codon is generated early on in the protein sequence by complete and/or partial deletion of 5’ exons corresponding to N-terminal domains of the protein (S1C Fig).

### Phenotypic characterization of the FOG-1 MEL knockout cells

The three selected FOG-1 KO MEL clones, D3, C8 and G9, were then subjected to phenotypic characterization by assessing their differentiation and hemoglobinization profiles. Knocking out FOG-1 in MEL cells results in defective DMSO-induced terminal differentiation, as suggested by morphological characterization. Specifically, May-Grünwald-Giemsa (MGG) staining shows that only DMSO-induced wild type (WT) MEL cells can differentiate to the polychromatic stage, with some cells even reaching the orthochromatic state (Fig 1B). By contrast, following DMSO induction of terminal differentiation, cells in the FOG-1 KO MEL clones remain in the proerythroblast stage (Fig 1B). Furthermore, benzidine staining showed a complete lack of hemoglobinization in the KO cells (Fig 1B). Additional evidence of defective DMSO-induced terminal differentiation in FOG-1 KO MEL cells, was obtained by flow cytometry using the erythroid cell surface marker CD44, which is extinguished during terminal erythroid differentiation [23]. As expected, CD44 extinction was observed in WT MEL cells, but not in DMSO-induced FOG-1 KO cells, though it is possible that CD44 expression is directly affected by the loss of FOG-1 (Figs 1C; S2A). In addition, propidium iodide (PI) staining was used to measure the percentage of proliferating cells in G2 phase, versus terminally differentiated cells which arrest in G1 phase. PI staining showed that an appreciable proportion of FOG-1 KO MEL cells continued to proliferate following DMSO induction, as evidenced by a higher number of KO cells in the G2 phase and, concomitantly, a lower number of cells in the G1 phase, compared to DMSO-induced differentiated WT MEL cells (Figs 1D; S2B). We also observed increased apoptosis (Figs 1E; S2C) and ROS levels (Fig 1F) in FOG-1 KO MEL cells during DMSO induction of differentiation. Considering GATA1’s anti-apoptotic functions [24] and antioxidant capacity [25] in erythropoiesis, these observations suggest that FOG-1 is also likely to be cooperating with GATA1 in fulfilling these functions.

### Expression profiling of FOG-1 knockout MEL cells by RNAseq

**Fig 2:**
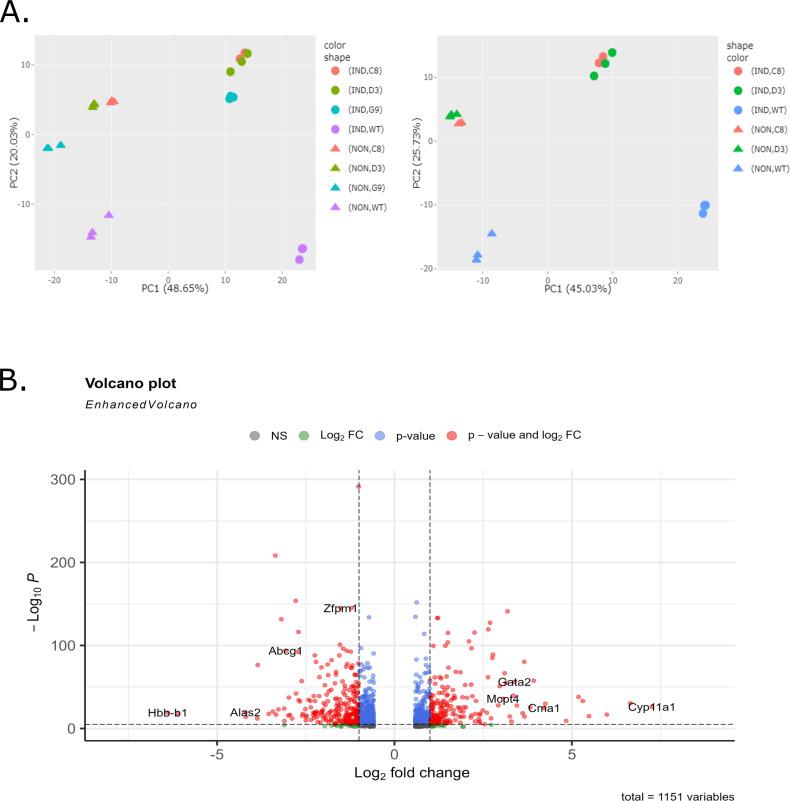
Principal Component Analysis (PCA). **(A) **The first two principal components are plotted for all samples (left panel) and for selected samples (right panel). Samples cluster according to genotype and induction status. In the left panel, the G9 FOG-1 KO clone clustered separately and was thus left out of subsequent differential gene expression analysis. Each point in the PCA plots represents an RNAseq replicate sample. Genotypes and induction status are indicated by using different colors and shapes as indicated in the legend provided. **(B)** Volcano plot of differentially expressed genes (DEGs) (>1.5-fold change, adjusted p-value < 0.01) between WT MEL and the C8 and D3 FOG-1 KO clones with induction status as a co-variate and selected representative genes labelled.

We next proceeded to carry out differential expression profiling by RNAseq of the FOG-1 KO MEL clones compared to WT MEL cells, before and after DMSO induction of differentiation. Principal component analysis (PCA) of the aligned sequences showed that the biological replicates of the FOG-1 knockout clones C8 and D3 clustered tightly together in both induced and non-induced samples, whilst G9 clustered independently (Fig 2A). Consequently, the G9 samples were excluded from downstream bioinformatic analysis, but were included in the functional validation work described below.

Comparison of bulk RNA expression between WT and the C8 and D3 FOG-1 knockout clones in uninduced and DMSO-induced MEL cells, showed differential expression (FC> ± 1.5, p < 0.01) in 1,151 genes (560 genes upregulated versus 590 down-regulated genes in wild type MEL versus FOG-1 knockout cells; S1 File) (Fig 2B). Down-regulated genes include, as expected, the *Zfpm1* gene, the erythroid *Hbb-b1* (βmajor) and *Hba-a1* globin genes and the *Alas2* heme biosynthesis gene (Figs 2B and S2). Upregulated genes include non-erythroid hematopoietic genes, such as the hematopoietic stem cell and megakaryocytic specific *Gata2* gene and the mast cell specific *Mcpt4* and *Cma1* genes (Figs 2B and S2).

**Fig 3:**
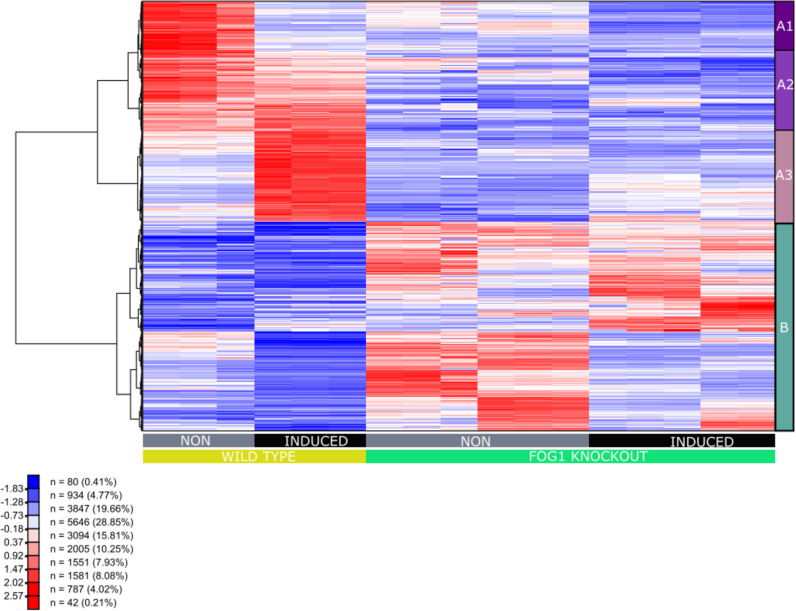
Heat map of transcription patterns of the wildtype MEL and the two FOG-1 knockout cell lines D3 and C8 displaying the expression profiles of the 1151 differentially expressed genes with greater than 1.5-fold change in expression and an adjusted p-value of < 0.01. Each row of the heat map represents a gene, each column represents a sample, and each cell displays normalized gene expression values, as indicated above. Groups of co-expressed genes (A1, A2, A3, B) identified by hierarchical clustering are marked to the right of the heatmap.

Hierarchical clustering divided differentially expressed genes (DEGs) into distinct groups, based on their relative expression profiles (Fig 3). Specifically, group A (591 genes, S1 File) includes genes that are expressed in non-induced WT cells but are not expressed, or are expressed at very low levels, in non-induced FOG-1 KO cells. In response to DMSO-induced differentiation in WT cells, these genes were either down-regulated (subcluster A1: 150 genes; S1 File), upregulated (subcluster A3: 242 genes, S1 File), or showed relatively unchanged expression (subcluster A2:199 genes, S1 File). By contrast, in FOG-1 KO cells, all group A genes are expressed at low levels in both non-induced and DMSO-induced MEL cells (Fig 3). Examples of the expression profiles of A1 genes (*Pld4* and *Havcr2*) and A2 genes (*Abcg1* and *Zfpm1*) are given in S2 Fig. Interestingly, A3 group genes include erythroid specific genes, such as *Hbb-b1, Hbb-a1* and *Alas2*, indicating that this group of genes represent the erythroid-specific transcription program (S2 Fig; see also below).

Group B includes 560 genes that are not expressed, or are expressed at very low levels, in both non-induced and DMSO-induced WT MEL type cells (Fig 3; S1 File). By contrast, these genes are generally expressed at higher, but variable, levels in non-induced and DMSO-induced FOG-1 KO cells, displaying complex patterns of differential expression (Fig 3). Hence, group B genes most likely represent genes that, in the absence of FOG-1, become de-repressed in non-induced cells and remain active, or become variably repressed upon DMSO induction of differentiation (Fig 3; S1 File). Examples include the mast cell specific *Cma1* and *Cpa3* genes which are de-repressed in non-induced cells in the absence of FOG-1, but become strongly repressed upon DMSO-induced differentiation, and the *Rps6ka2* and *Grin2d* genes that are derepressed in non-induced cells in the absence of FOG-1, remaining active in DMSO-induced cells (S2 Fig). *Klf10* is an example of a gene that becomes more upregulated upon DMSO induction in the FOG-1 KO cells, compared to the wild type cells (S2 Fig). Interestingly, the *Gata2* and *Gata3* genes also belong to the B group of genes (S1 File), as they become derepressed in non-induced FOG-1 KO cells and remain active, albeit at lower levels, in the induced FOG-1 KO cells (S2 Fig). By contrast, as also seen previously with the *Zfpm1* knockout in mice [3], *Gata1* expression is not significantly affected in the FOG-1 KO cells (S2 Fig) suggesting that (i) *Zfpm1* is downstream of GATA1, and (ii) the phenotypic effects we observe in the FOG-1 KO MEL cells are not due to deregulation of *Gata1* expression.

### Gene ontology (GO) and pathway analysis of differentially expressed genes

**Fig 4:**
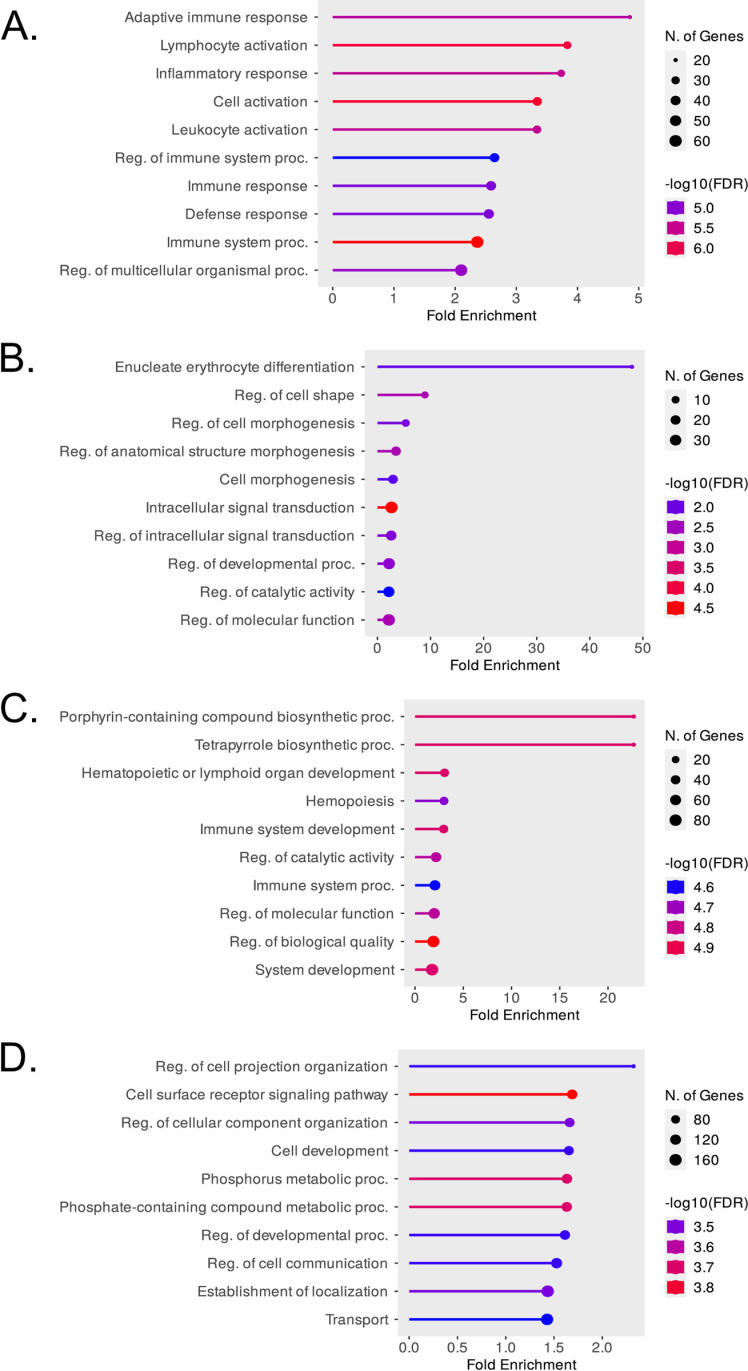
Gene Ontology (GO) Biological Process analysis of genes in clusters A1 (panel A), A2 (panel B), A3 (panel C) and B (panel D) of the hierarchical clustering analysis in Fig 3. The top 10 GO Biological Process terms are shown in each case.

Analysis of the differentially expressed gene sets in subclusters A1 to A3 in the FOG-1 KO cells against curated databases using ShinyGO (0.77) [26], showed that group A genes were enriched for GO Biological Process terms associated with hemato/lymphopoiesis and erythroid development (Fig 4). Specifically, A1 genes were enriched for GO Biological Process terms associated with lymphoid cells and immune-related functions (Fig 4A), indicating that these genes are expressed in non-induced MEL cells but become repressed upon terminal differentiation of MEL cells. Interestingly, the expression profiles of A1 genes in the FOG-1 KO MEL cells suggest that, in the absence of FOG-1, they fail to be expressed in non-induced MEL cells (Fig 3), even though the lymphoid-specific GATA3 transcription factor is expressed in non-induced FOG-1 KO MEL cells (S2 Fig). The precise nature of a potential involvement for FOG-1 in lymphoid-related gene expression in WT non-induced MEL cells is unclear but warrants further investigation. For A2 subcluster genes, the most highly enriched GO Biological Process term is related to erythroid differentiation, whereas other enriched terms relate to broader cell signaling, developmental and cellular properties and functions (Fig 4B). Again, expression profiles of A2 genes in the FOG-1 KO MEL cells suggest that FOG-1 may be required for their expression, particularly in non-induced MEL cells (Fig 3). Subcluster A3 genes are enriched for erythroid-related and hematopoietic GO Biological Process terms (Fig 4C), confirming that this subcluster most likely represents the erythroid-specific transcription program that fails to be upregulated with differentiation upon DMSO induction (Fig 3). Analysis of B cluster genes shows an enrichment for GO Biological Process terms related to cellular functions, signaling and metabolism (Fig 4D).

Transcription factor target gene enrichment analysis using the ChIP Enrichment Analysis database (ChEA) [27] in ShinyGO, showed that A cluster genes were enriched for GATA1 and TAL1 binding (S3A Fig), whereas ChEA analysis of B cluster genes showed an enrichment for targets genes of the megakaryocytic FLI1 and RUNX1 transcription and for the myeloid PU.1 transcription factor (S3B Fig). KEGG pathway analysis showed an enrichment for pathways associated with platelets and inflammation for genes in subcluster A1 (S4A Fig), pathways associated with infection for genes in subcluster A2 (S4B Fig) and metabolic pathways, including porphyrin metabolism, for genes in subcluster A3 (S4C Fig). KEGG pathway analysis of B cluster genes showed an enrichment for signaling and metabolic pathways (S4D Fig).

**Fig 5:**
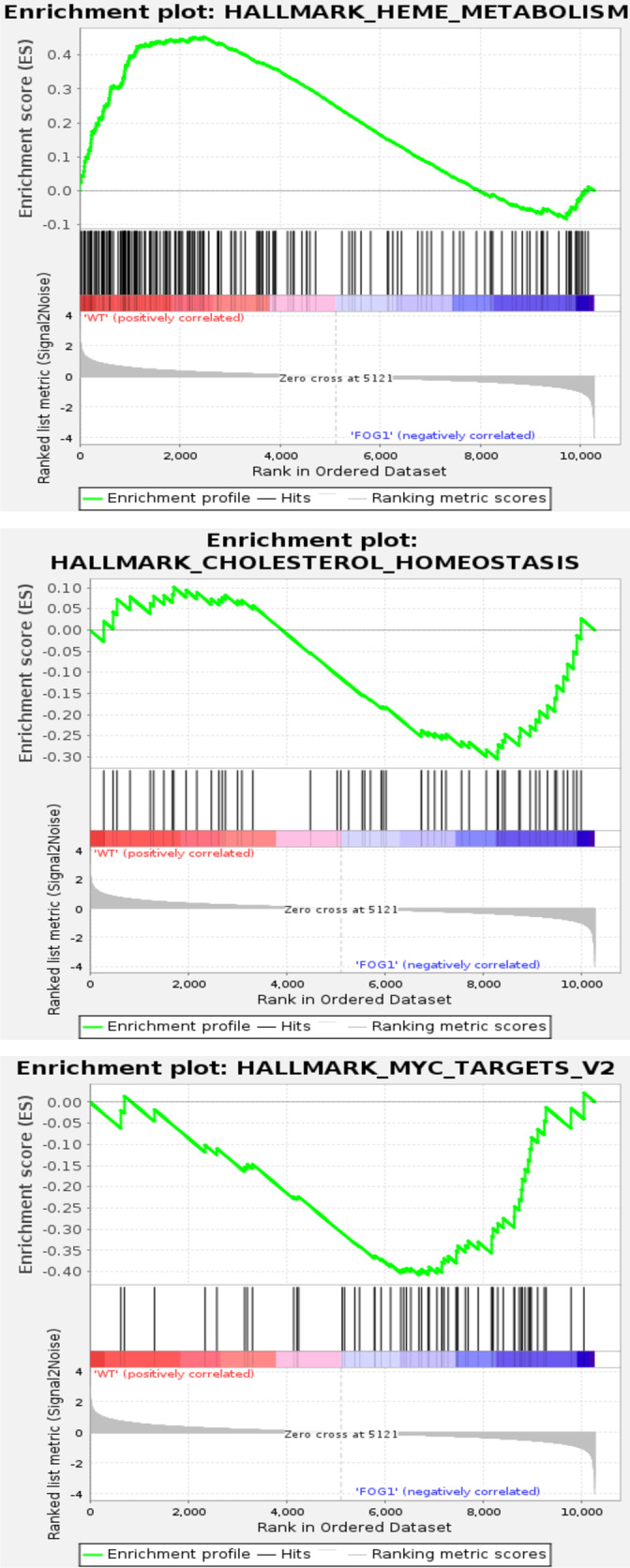
GSEA analysis showing enrichment of gene sets for Heme metabolism, Cholesterol homeostasis and Myc targets.

We next carried out GSEA analysis of WT MEL cells versus the FOG-1 KO against the MSig database [28,29]. This showed that gene sets for heme metabolism, cholesterol homeostasis and Myc targets were strongly overrepresented (Figs 5 and S5). A closer look at the expression profiles of genes in the Heme Metabolism Pathway showed that genes become inversely (de)regulated in the FOG-1 KO cells compared to WT cells. For example, genes that are expressed in WT cells, fail to express in FOG-1 KO cells, or genes that are not expressed in WT cells, become de-repressed in the FOG-1 KO cells (S5 Fig). Similarly, when inspecting the expression profiles of genes in the Cholesterol Homeostasis gene set, we see evidence of genes becoming de-repressed in the non-induced FOG-1 KO cells compared to WT cells, or of genes that are not active in non-induced cells and/or in DMSO-induced FOG-1 KO cells, compared to WT cells (S5 Fig). Interestingly, inspection of the expression profiles of the Myc Target Genes Pathway shows incomplete repression in DMSO-induced FOG-1 KO cells, in sharp contrast to WT MEL cells which show a strongly repressed profile upon DMSO induction (S5 Fig). It is known that GATA1 represses *c-Myc* expression directly [30] and indirectly [31], with GATA1-mediated *c-Myc* repression being a requirement for proliferation arrest during erythroid terminal differentiation [30]. Our observations suggest that FOG-1 plays a role in the GATA1-mediated repression of *c-Myc* and its downstream gene targets, warranting further investigation.

### FOG-1 reguvlation of cholesterol transport

**Fig 6:**
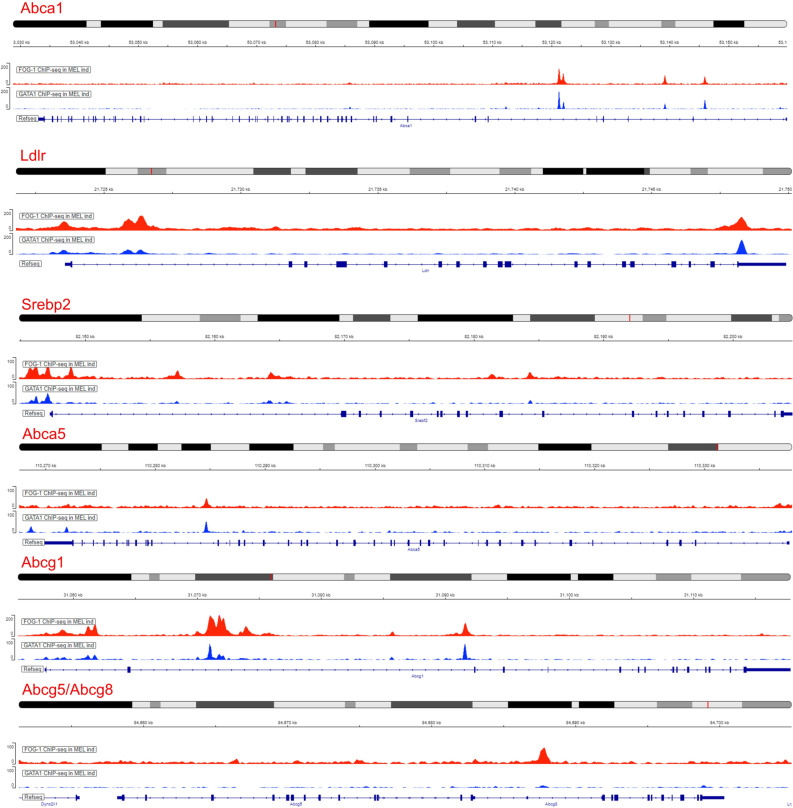
GATA1 and FOG-1 occupancies by ChIPseq of the *Abca1, Abcg1, Abcg5/8, Abca5, Ldlr* and *Srebf2* genes in DMSO-induced MEL cells. Interestingly, the shared promoter of the *Abcg5 and Abcg8* genes is occupied only by FOG-1. GATA1 MEL/DMSO ChIPseq dataset used: ENCSR000ETA. FOG-1 MEL/DMSO ChIPseq dataset: SRA ID PRJNA1198023.

The KEGG pathway analysis highlighted ABC transporters as being enriched primarily in A3 cluster genes (S4 Fig). Differentially regulated ABC transporter genes in the FOG-1 KO MEL cells include *Abca1, Abca4, Abca5, Abcb6, Abcb9, Abcb10* and *Abcg1* (S1 File). Of those, *Abcb10* (also known as ABC-me) and *Abcb6* are known to have functions in erythropoiesis. Specifically, *Abcb10* is a GATA1-regulated gene [32] known to play an essential role in heme biosynthesis [33], whereas *Abcb6* is a known mitochondrial porphyrin transporter [34]. *Abcb9* is a lysosomal peptide transporter [35] and *Abca1*, *Abca5* and *Abcg1* are cholesterol transporters associated with cholesterol efflux [36]. Interestingly, cholesterol homeostasis was enriched in the GSEA analysis (Fig 6) and together with the differential expression of ABC cholesterol transporters in the FOG-1 KO cells, suggest a novel function for FOG-1 (and, most likely, GATA1) in cholesterol homeostasis, potentially through regulation of cholesterol transport. Hence, we next focused on investigating the involvement of FOG-1 in cholesterol transport in greater detail. Utilising FOG-1 and GATA1 ChIP-seq data in differentiated WT MEL cells [37], we assessed the GATA1 and FOG-1 occupancies of cholesterol transporter genes (Fig 6). For completeness, we also included in our analysis the *Abcg5/Abcg8* and *Ldlr* cholesterol transporters. By comparing ChIPseq profiles, it is evident that a number of cholesterol transporter genes regulatory regions are occupied by both GATA1 and FOG-1 (Fig 6). We also interrogated histone modification marks in ChIPseq data from DMSO-induced MEL chromatin, namely, histone H3 lysine 4 trimethylation (H3K4me3, associated with active or poised promoters) and histone H3 lysine 4 monomethylation (H3K4me1, associated with enhancers and with DNA regions downstream of transcription start sites), as well as occupancies of RNA polymerase II (RNAPol II). From these it can be seen that FOG-1/GATA1 binding concoides mostly with intragenic sequences that may serve as enhancers (*Abca1, Ldlr, Abca5, Abcg1)*, and less frequently with promoters (*e.g., Ldlr* and *Srebf2*, see below)(S6 Fig). Of interest, FOG-1 appears to bind to a sequence in the *Abcg8* gene in the absence of GATA1 binding (Fig 6). The significance of this observation is unclear but warrants further investigation.

**Fig 7:**
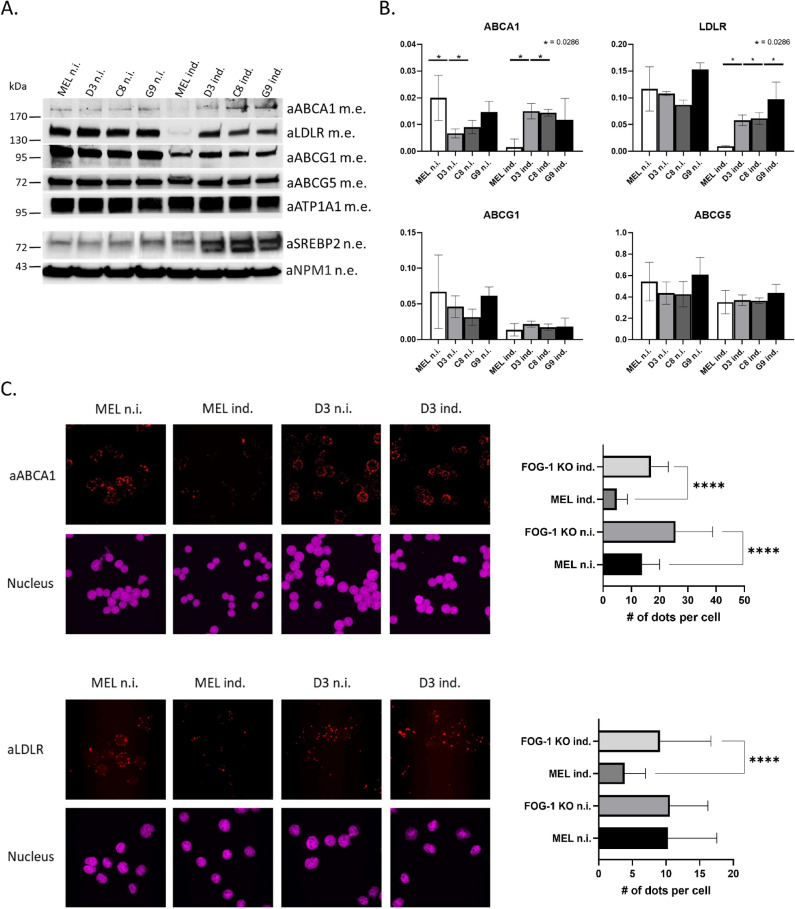
(A) Western Blot analysis of ABCA1, ABCG1, ABCG5/G8, LDLR and SREBP2 proteins in non-induced and DMSO-induced WT and FOG-1 KO MEL cell clones D3, C8 and G9. Cell membrane protein extracts were used for the analysis of cholesterol transporters and nuclear extracts for the analysis of SREBP2. ATP1A1 (ATPase Na+/K+ transporting subunit alpha 1) and NPM1 (nucleophosmin 1) were used as protein loading controls for cell membrane and nuclear extracts, respectively. **(B)** Quantification of ABCA1, ABCG1, ABCG5/G8 and LDLR protein levels in non-induced and DMSO-induced WT and FOG-1 KO MEL cell clones D3, C8 and G9 using cell membrane protein extracts (n = 4). **(C)** Left panels: Immunofluorescence analysis of ABCA1 (top panels) and LDLR (lower panels) expression in non-induced and DMSO-induced WT and FOG-1 KO clone D3 cells. Cell nuclei were counterstained with DAPI. Right panels: quantitation (number of speckles per cell) of the immunofluorescence images for ABCA1 and LDLR shown in the left panels. ****p < 0.0001 as obtained by t-test compared to signal from WT MEL cells. n.i.: non-induced cells; ind.: DMSO-induced cells.

Guided by the ChIPseq analysis, we next assessed the protein levels of the ABCA1, ABCG1, ABCG5, ABCG8 and LDLR cholesterol transporters in cell membrane protein extracts by Western blot analysis (WB). From this, it is clear that the ABCA1 and LDLR cholesterol transporters, which are bound by FOG-1 and GATA1, are deregulated in the FOG-1 KO cells (Fig 7A). Specifically, in WT MEL cells, ABCA1 and LDLR levels are repressed with erythroid differentiation, whereas in DMSO-induced FOG-1 KO cells, ABCA1 and LDLR protein levels remain high (Fig 7B). This observation was further validated by immunofluorescence, which confirmed the higher ABCA1 and LDLR protein levels in the cell membrane of FOG-1 KO MEL cells, compared to WT MEL cells, following induction by DMSO (Fig 7C). Taken together, these observations suggest that, in the absence of FOG-1, expression of ABCA1 and LDLR proteins fails to become repressed upon DMSO induction. By contrast, expression of the ABCG1 and ABCG5 cholesterol transporters is unaffected in the FOG-1 KO MEL cells (Fig 7A, B), despite the fact that the former apears to be bound by GATA1 and FOG-1 (Fig 6).

### SREBP2 and cholesterol transport

We next turned our attention to SREBP2, a known transcriptional regulator of the *Abca1* and *Ldlr* genes [38,39] and of cholesterol biosynthesis and homeostasis in general [40]. Interestingly, recent work showed a functional interaction between GATA1 and SREBP2 proteins in erythroid cells, whereby GATA1 binds to SREBP2 to downregulate cholesterol biosynthesis, leading to a gradual reduction in intracellular cholesterol levels [41]. ChIPseq data in MEL cells suggest that the promoter of the *Srebf2* gene, which codes for SREBP2, is occupied by GATA1 and FOG-1 binding primarily to its promoter region (Figs 6 and S6). Although expression of *Srebf2* does not appear to be significantly affected in FOG-1 KO cells (S1 File), the aforementioned observations of a SREBP2/GATA1 functional interaction, the possibility of erythroid-specific regulation of *Srebf2* by GATA1 and FOG-1, and the known transcriptional regulatory function of SREBP2 in regulating the *Abca1* and *Ldlr* genes genes, prompted us to examine in greater detail the expression of SREBP2 in FOG-1 KO MEL cells. Western immunoblot analysis showed that, indeed, nuclear SREBP2 protein levels are increased in FOG-1 KO cells under DMSO induction, compared to induced WT cells (Fig 7A). Nuclear SREBP2 protein is detected as a ~70kDa band in nuclear extracts from MEL cells, with a second lower band detected in DMSO-induced FOG-1 KO cells, which most likely corresponds to a cleaved degradation product [42]. These observations raise the possibility that LDLR and ABCA1 protein levels in DMSO-induced FOG-1 KO cells may be the combination of a direct effect of the loss of FOG-1 acting through GATA1 and of an indirect effect through the upregulation of SREBP2 in the absence of FOG-1. By contrast, the known SREBP2-regulated cholesterol biosynthesis *Hmgcs1* and *Hmgcr* genes did not show reproducible changes in their protein levels in FOG-1 KO cells (S7 Fig).

### Intracellular cholesterol levels and membrane fluidity in FOG-1 KO cells

**Fig 8:**
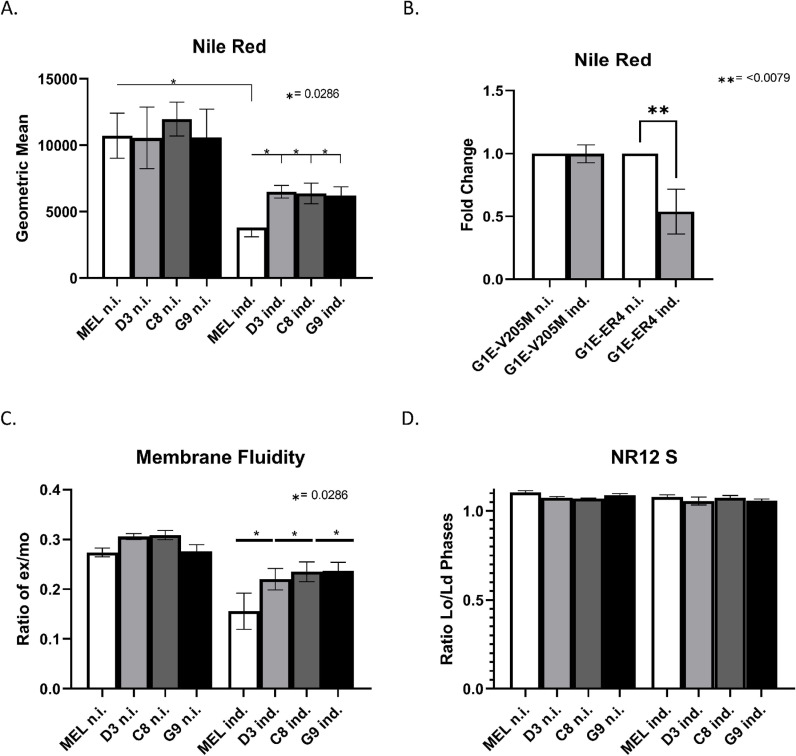
(A) Quantitation of Nile Red staining of intracellular lipid droplets and neutral lipid levels during DMSO-induced differentiation of WT and FOG-1 KO MEL cell clones C3, C8 and G9. **(B)** Quantitation of Nile Red staining of intracellular lipid droplets and neutral lipid levels during differentiation of the G1E cell line derivatives G1E-V205M and G1E-ER4 (see the Results section for more information on G1E cells). **(C)** Membrane fluidity assay during DMSO-induced differentiation of WT and FOG-1 KO MEL cell clones C3, C8 and G9. The data is plotted as the ratio between pyrene excimer and monomer (ratio Ie/Im). The higher the ratio, the more fluid (or less rigid) the membrane is. **(D)** Quantitation of plasma membrane cholesterol levels by NR12S staining in DMSO-induced differentiation of WT and FOG-1 KO MEL cell clones C3, C8 and G9. Four biological replicates were done for all the above assays. n.i.: non-induced cells; ind.: DMSO-induced cells.

The lack of repression of the ABCA1 and LDLR cholesterol transporters in FOG-1 KO MEL cells under DMSO induction, may lead to alterations in intracellular lipid droplet levels in the KO cells, which in turn might have an overall effect on cellular properties such as membrane fluidity. To test this, we used Nile Red to stain for intracellular lipid droplets (esterified cholesterol). This showed a significant decrease of intracellular cholesterol levels during DMSO induced differentiation of WT MEL cells (Fig 8A), in agreement with previous studies [43]. By contrast, the reduction in intracellular cholesterol levels upon DMSO induction was less pronounced in FOG-1 KO cells (Fig 8A). This suggests that the net effect is an increase in intracellular lipid levels.

Next, we tested whether the interaction of FOG-1 with GATA1 is necessary for the reduction of intracellular cholesterol levels during erythroid differentiation. To do this, we used the G1E GATA1 null murine prerythroblastic cell line [44]. Restoration of β-estradiol-inducible expression of GATA1 (G1E-ER4), allows G1E cells to complete erythroid differentiation. Moreover, inducible expression in G1E cells of the V20M GATA1 mutant (G1E-V205M) which abolishes interaction with FOG-1, allows the dissection of GATA1/FOG-1 specific functions in erythropoiesis [13]. We stained G1E-ER4 and G1E-V205M cells with Nile Red and saw a 2-fold reduction of intracellular cholesterol levels upon induction of GATA1 expression by β-estradiol in G1E-ER4 cells (Fig 8B). By contrast, there was little change in intracellular cholesterol levels in the induced G1E-V205M cells, suggesting that the physiological reduction in intracellular cholesterol levels observed in erythropoiesis, requires a functional GATA1/FOG-1 interaction.

Next, we used a membrane fluidity assay that utilizes the properties of pyrenedecanoic acid (PDA). PDA incorporates into the cell’s plasma membrane forming either monomers or excimers, with the rate of excimer formation being proportional to the membrane fluidity. Using this assay, we tested whether the increased intracellular cholesterol levels in the DMSO-induced FOG-1 KO cells had an effect on the cell membrane properties of the cells. We found that the FOG-1 KO cells showed a small, but significant, increase in membrane fluidity compared to WT MEL cells under DMSO induction (Fig 8C). To assess whether the increased membrane fluidity is attributed to higher levels of cholesterol within the plasma membrane, we stained WT and KO cells with the Nile Red 12S (NR12S) dye which measures lipid order changes in the outer membrane leaflet. The fluorescence emission spectra of the NR12S changes in response to lipid order, shifting towards shorter wavelengths when incorporated into a liquid ordered (Lo) phase (i.e., increased cholesterol), compared to a liquid disordered phase (Ld). The Lo phase was measured with the YG586 filter and the Ld phase was measured with the YG670 filter. Interestingly, this assay showed that cholesterol levels in the outer plasma membrane leaflet remain the same regardless of DMSO induction, or cell condition (KO versus WT; Fig 8D). Further investigation is needed to assess in greater detail any changes in the cell membrane of FOG-1 KO cells during erythroid differentiation, for example, by cell membrane lipidomics.

## Discussion

FOG-1 is a key transcriptional co-factor of GATA1 in regulating erythropoiesis. The *Zfpm1* gene knockout in mice has shown FOG-1 to be essential for erythropoiesis, presenting with an erythroid phenotype that is very similar to that of the *Gata1* gene knockout [3]. Despite the publication of the *Zfpm1* knockout in 1998, little is known about the molecular basis of the phenotype in erythroid cells. Here, we addressed this by employing CRISPR/Cas9 gene editing to knockout the *Zfpm1* gene in MEL cells, an *in vitro* cellular model of murine erythropoiesis. The phenotypic analysis, as expected, showed an arrest in DMSO-induced erythroid differentiation of FOG-1 knockout cells at the proerythroblastic to orthochromatic stages and a lack of hemoglobinization. In addition, we saw a small (not significant, except for knockout clone D3) increase in apoptosis, an incomplete cell cycle arrest and increased ROS levels upon DMSO-induced differentiation. Thus, our phenotypic analysis of the FOG-1 knockout MEL cells, complements and extends that of the *Zfpm1* knockout mice [3].

To explore further the molecular basis of the FOG-1 knockout phenotype, we carried out expression profiling of the FOG-1 knockout cells by RNAseq. Consistent with the phenotypic analysis, we found a failure of the upregulation of the erythroid transcription program upon DMSO induction and an incomplete repression of early hematopoietic (e.g., *Gata2*) genes and of genes belonging to alternative hematopoietic lineages, such as the mast cell-specific genes *Cma1*, *Cpa3* and the lymphoid-specific *Gata3* gene (Figs 2 and S2). The case of the mast cell-specific *Cma1* and *Cpa3* genes is of interest. FOG-1 is not expressed in mast cells [2], in contrast to GATA1 and GATA2 which are both expressed and play key roles in mast cell lineage determination [45]. Importantly, ectopic expression of FOG-1 in mast cell progenitors reprograms them towards erythroid, megakaryocytic and granulocytic lineages, providing strong evidence for an antagonistic role for FOG-1 in supressing the mast cell lineage [46,47]. Hence, the derepression of the *Cma1* and *Cpa3* genes we see in non-induced FOG-1 KO MEL cells (S2 Fig) is consistent with FOG-1’s documented mast cell repressive function. Interestingly, the *Cma1* and *Cpa3* derepression in non-induced FOG-1 knockout MEL cells, which are committed proerythroblasts, suggests that the transcription factor environment in the FOG-1 knockout MEL cells can support *Cma1* and *Cpa3* transcription. For example, it is conceivable that the high levels of derepressed GATA2 in non-induced FOG-1 knockout MEL cells together with GATA1, which remains unaffected by the FOG-1 knockout (S2 Fig), may be sufficient to support *Cma1* and *Cpa3* expression. Alternatively, or in addition, an increase in the expression of the mast cell specific *Mitf* transcription factor seen in FOG-1 KO MEL cells (S2 Fig), may also contribute to the expression of mast cell specific genes. Interestingly, *Cma1* and *Cpa3* expression is extinguished in DMSO-induced FOG-1 knockout MEL cells (S2 Fig), despite the fact that these cells are incapable of completing erythroid differentiation. This loss may be due to the reduction in GATA2 expression observed in the DMSO-treated FOG-1 knockout MEL cells (S2 Fig) and/or the loss or repression of other factors that are required to support *Cma1* and *Cpa3* expression, such as *Mitf* which is moderately down-regulated in FOG-1 DMSO-induced MEL cells (S2 Fig). It would also be of interest in the future to investigate the molecular basis of the reduction in GATA2 (and GATA3) expression observed in DMSO-induced FOG-1 knockout MEL cells (S2 Fig). FOG-1 cell lineage suppression functions have also been documented in other hematopoietic lineages, for example, in eosinophils [48]. However, the molecular basis of the lineage-repressive functions of FOG-1 remains poorly understood. The FOG-1 knockout MEL cell lines described here present with a very useful tool for exploring the FOG-1 lineage repressive functions, for example, by functional complementation assays using transfected FOG-1 deletion, or other, mutants.

We also describe an enrichment for ABC transporters and cholesterol homeostasis in our pathway and gene set enrichment analysis, respectively, thus linking FOG-1, and consequently GATA1, to the regulation of cholesterol transport and homeostasis in erythroid cells. Specifically, we found that ABC transporter genes involved in cholesterol transport were differentially expressed, with several of them being occupied by GATA1 and FOG-1 in DMSO-induced MEL cells, as evidence by ChIPseq data (Figs 6 and S6). We validated the ABCA1 and LDLR cholesterol transporters as being differentially expressed in the FOG-1 knockout MEL cells, in that they failed to be completely repressed upon DMSO induction, compared to wild type cells. The sustained levels of ABCA1 and LDLR proteins are also reflected by higher levels of intracellular lipid droplet in DMSO-induced FOG-1 knockout cells. This accumulation, in turn, may exert a broader influence on the mechanical attributes of the cell membrane, such as increased fluidity. It is possible that the sustained levels of ABCA1 and LDLR expression and subsequent effects on intracellular lipid droplets and membrane fluidity, are due to the incomplete differentiation of the DMSO-induced FOG-1 knockout cells. However, the fact that the ABCG1 and ABCG5 cholesterol transporters decline in the FOG-1 KO cells to the same extent as in wild type cells during differentiation, suggests that the effects we see with ABCA1 and LDLR are not due to arrested MEL cell differentiation in the KO cells. If anything, the ABCA1 protein levels increase slightly with differentiation in the FOG-1 KO cells, again arguing for a specific effect rather than a general differentiation arrest defect. Future experiments abrogating FOG-1 and GATA1 binding to the *Abca1* and *Ldlr* gene loci by gene editing will establish the erythroid-specific regulation of the two genes by GATA1 and FOG-1 and will help elucidate their function in cholesterol transport and homeostasis in erythropoiesis.

We also show that nuclear protein levels of SREBP2, a transcription factor regulating cholesterol biosynthesis and transport, are significantly upregulated in DMSO-induced FOG-1 knockout cells, compared to wild type cells (Fig 7). SREBP2 levels do not change much with DMSO-induced differentiation in wild type MEL cells (Fig 7). By contrast, there is a marked upregulation of SREBP2 in induced FOG-1 knockout cells (Fig 7), suggesting a specific deregulation of SREBP2 in the absence of FOG-1. We note also that GATA1 and FOG-1 bind to the promoter of the *Srebf2* gene which codes for SREBP2 (Figs 6 and S6). Taken together, our observations suggest that GATA1 and FOG-1 binding to *Srebf2* serves to keep SREBP2 levels low during MEL cell differentiation. In addition, our observations suggest that the observed changes in ABCA1 and LDLR may be a direct effect of the loss of FOG-1 binding to their corresponding genes (Figs 6 and S6), as well as an indirect effect due to derepressed SREBP2 acting on the *Abca1* and *Ldlr* genes in DMSO-induced FOG-1 knockout cells. Lastly, it was recently reported that GATA1 protein physically interacts with SREBP2 to inhibit the latter’s function in activating cholesterol biosynthesis genes during erythroid differentiation [29]. Our findings add to the observations of Lu et al. implicating FOG-1, together with GATA1, in suppressing SREBP2 functions in cholesterol homeostasis at the transcriptional level [41]. Moreover, our findings add another layer to the GATA1/FOG-1/SREBP2 cholesterol regulatory axis by suggesting that GATA1 and FOG-1 act directly on the *Srebf2* gene in erythroid cells. Overall, our observations with the FOG-1 knockout cells are consistent with those of Lu et al. regarding the cholesterol-repressive functions of GATA1 in erythroid differentiation [41].

In a broader context, our observations and those of Lu et al. [41] raise the question as to why cholesterol levels need to be tightly regulated by GATA1 and FOG-1 in terminal erythroid differentiation. Cholesterol is a key factor regulating the membrane fluidity and stability of cells. This is of particular relevance to red blood cells, since membrane fluidity allows the cells to adapt to various conditions, at the same time preventing them from becoming too rigid or permeable and providing them with the ability to withstand the mechanical stress of going through smaller blood vessels and capillaries [49]. Cellular cholesterol levels are determined by the interplay of *de novo* biosynthesis, uptake, export and esterification [50]. Although all mammalian cells can produce cholesterol, most (except for hepatocytes, adrenal cells and gonadal cells) are unable to catabolize it and need to dispose excess cholesterol out of the cell, or store it as cholesteryl esters in lipid droplets [51]. Therefore, reverse cholesterol transport and inhibiting cholesterol absorption from plasma are important in regulating cellular levels of cholesterol.

Previous studies have provided evidence for cholesterol homeostasis being an important factor in hematopoiesis [52,53]. However, the molecular basis of cholesterol homeostasis in erythropoiesis remains largely uncharted. To-date there have been a handful of publications investigating the effects of cholesterol homeostasis in erythroid differentiation, focusing mainly on cholesterol biosynthesis [54–56] and cholesterol efflux [52,57–60]. Evidence from early studies in MEL cells in the 1980s, showed a major decrease in cholesterol content during erythroid maturation [43]. Subsequent studies showed that an excess of cellular cholesterol levels could block MEL differentiation [41,61], suggesting that cholesterol levels play an important role in erythropoiesis. More recent in vitro and in vivo studies raised the possibility that cholesterol biosynthesis regulates the differentiation of red blood cells (RBCs), as cholesterol synthesis-related enzymes and their regulators are required to maintain self-renewal of primary erythroid progenitors [55,62]. In addition, recent studies suggested an active role for RBCs in reverse cholesterol transport in the circulation. For example, a study by Ohkawa et al. [63] indicated that the movement of cholesterol between the red blood cell and plasma is saturable and energy, temperature and time-dependent, suggesting an active transport mechanism involving cholesterol transporters. Interestingly, recent studies showed a direct [63] and indirect [64] involvement of ABCA1 in cholesterol influx, which is contrary to the established notion of ABCA1 functioning as a cholesterol exporter. Hence, our observations of increased lipid droplet levels in induced FOG-1 knockout cells could be the result of the combined effect of ABCA1 and LDLR acting on cholesterol influx. Overall, it is evident that the regulation of cholesterol homeostasis during erythroid differentiation is important for the specialized physical properties of mature RBCs and their role in reverse cholesterol transport in the circulation. Our observations suggest that the fine-tuning of cholesterol homeostasis in erythropoiesis is, at least in part, under the control of GATA1 and its co-factor FOG-1.

Our study also presents with limitations. For example, the different *Zfpm1* gene edits in the three clones (S1 Fig) and possible off-target effects, may account for variability in the expression profiling patterns, e.g., in clone G9. Also, the lack of a commercially available ChIP-grade FOG-1 antibody, precluded us from verifying FOG-1 binding to the cholesterol transport and other potential target genes. MEL cells also present with limitations as they do not fully recapitulate murine terminal erythroid differentiation. Nevertheless, our findings suggest that FOG-1 plays a direct and/or indirect role, possibly mediated through SREBP2, in the repression of cholesterol transporters that may be necessary for the progression of erythroid differentiation. Additional work is needed to explore this in greater detail. Future studies will focus on providing conclusive evidence for FOG-1 and GATA1 regulating the expression of cholesterol transporters, for example, by using gene editing to abrogate GATA1 and FOG-1 binding to ABC cholesterol transporter genes and the *Ldlr* gene, also in more relevant cellular models such as primary proerythoblasts from mouse fetal liver or bone marrow*.* Lastly, the generation of the FOG-1 knockout MEL cell lines and their transcriptomic profiles described here, set the stage for future work to unveil the broader role of FOG-1 in erythropoiesis beyond the pathways touched on here.

## Materials and Methods

### Cell lines

Mouse erythroleukemic (MEL) C88 cells (RRID:CVCL_C188) were cultured and induced to differentiate with 2% dimethylsulfoxide (DMSO) for 4 days, as previously described [65]. G1E, G1E-ER4 and G1E-V205M-ER4 cells [44,66] were a kind gift from Gerd Blobel (The Children’s Hospital of Philadelphia, University of Pennsylvania, Philadelphia, USA). These cells were cultured and induced with β-estradiol to express GATA1, as previously described [44,66].

### FOG-1 knockout MEL cells

The murine *Zfpm1* gene knockout (KO), coding for FOG-1, was performed by CRISPR/Cas9 in MEL cells with a commercially available kit designed to disrupt a gene by causing a double-strand break (DSB) in a 5’ constitutive exon. The FOG-1 CRISPR/Cas9 KO kit (Santa-Cruz, #sc-423807) consisted of a pool of 3 plasmids, each encoding the Cas9 nuclease and a target-specific 20 nucleotide guide RNA (gRNA) derived from the Genome-scale CRISPR Knock-Out (GeCKO) v2 library [67]. The sequences of the gRNAs included with the kit are not disclosed by the manufacturer. For the transfection, 2.5 x10^5^ MEL cells were aliquoted in a 6-well plate containing 3 ml culture medium without antibiotics and cultured for 24 hours. The next day, the transfection solution (10 μl of CRISPR/Cas9 plasmid, 10 μl of transfection reagent and 280 μl of transfection medium) was prepared and incubated for 25 minutes at room temperature prior to adding to the cultured cells. Transfected MEL cells were then cultured further for 1 week under G418 selection prior to GFP single cell sorting by FACs, (GFP is expressed from the Cas9/gRNA plasmid) and processing for downstream experiments.

### RNA isolation and generation of Illumina library for NGS

RNA was isolated from 10^7^ cells using the RNAeasy Mini Kit (QIAgen, #74104), following the manufacturer’s protocol. Samples were DNase Q treated and assessed for RNA integrity using the Agilent Bioanalyzer. Samples with RNA integrity (RIN) scores of 9.0 or higher were used to make a cDNA library using the TruSeq Stranded mRNA from Illumina (#20020594), following the manufacturer’s protocol. 1500ng of total RNA was used as input into the TruSeq Stranded mRNA kit (Illumina) as per the manufacturer’s protocol, with the following adjustments. Samples were fragmented at 94°C for 3 min, cDNA was reverse transcribed using Superscript IV (ThermoFisher, #12594025) under the following conditions: 25°C for 10 minutes, 50°C for 12 minutes and 80°C for 10 minutes. PCR was performed under the following conditions: 98°C for 30 seconds, followed by 9 cycles of 98°C for 10s/60°C for 30s and 72°C 35s, followed by 72°C for 5 minutes and hold at 10°C. Libraries were quantified using the Quant-iT PicoGreen dsDNA Assay Kit (ThermoFisher, #P11496), and a subset of libraries were validated with the Bioanalyzer 2100 using a DNA1000 chip (Agilent). Libraries were pooled in equimolar amounts and sequenced using the MGISeq2000 platform (BGI).

### RNAseq analysis

Raw sequencing reads (57.3 +/− 8.3 million reads per sample) were quality controlled, deduplicated, trimmed and filtered using Trimmomatic [68] and BBTools (https://sourceforge.net/projects/bbmap/). Samples were aligned to the Ensembl GRCm38 mouse genome (mean aligned reads 80.4% ± 1.7%) using Salmon (1.6.0) [69]. Hierarchical clustering and PCA analysis revealed no significant batch effects between replicates, hence technical replicates were combined for further analysis. Transcript level estimates of gene expression were imported into DESeq2 using tximeta [70]. Differential expression analysis was conducted using DESeq2 (1.42.0) in R via the interactive analysis and visualization tool DEBrowser (1.30.0) [71]. Differential expression analysis was based on genotype with induction status as a covariate. Differentially expressed genes were determined using the following thresholds: log2 (fold-change) ≥ 1.5 or ≤−1.5 and p value < 0.01. Clusters of differentially expressed genes were identified using the clusterProfiler package (4.10.0) [72] and the Next-Generation (Clustered) Heat Maps V2 program (https://bioinformatics.mdanderson.org/public-software/ngchm/). Gene Set Enrichment Analysis (GSEA) was conducted on the full normalized counts matrix against the orthology-mapped hallmark gene sets (MH) using the stand-alone GSEA program (4.3.2) with the MSigDB 2022.1 database. Gene ontology and KEGG analysis plus RNA-Seq and ChIP-Seq database comparisons were done using ShinyGO (0.77) [26]. The RNAseq data are accessible through the Sequence Read Archive (SRA) with ID number: PRJNA1172098. The FOG-1 ChIPseq data are accessible through SRA with ID: PRJNA1198023.

### May-Grünwald/Giemsa (MGG) staining

For MGG staining, 10^5^ cells were initially spun at 700 rpm for 10 minutes onto glass slides using the Cytospin 3 centrifuge (Shandon). The glass slides were stained using May-Grünwald (Sigma-Aldrich, #MG500)/Giemsa stain (Sigma-Aldrich, #GS500) according to manufacturer’s instructions. Pictures were captured using a Rebel (Echo REB-01-D) brightfield microscope at 40x magnification.

### Benzidine staining

One part of benzidine reagent solution (Sigma-Aldrich, #D-9143) was added to 10 parts of cell culture and after a 2-minute incubation, 10^5^ cells were used for cytospin followed by MGG staining, as above. Pictures from the slides were captured using a Rebel (Echo REB-01-D) brightfield microscope at 40x magnification.

### Nuclear and membrane protein extracts

Nuclear proteins from all cell types were prepared using the NUN method [73]. Membrane extracts were prepared using the Mem-PER Plus Membrane Protein Extraction Kit (ThermoFisher, #89842) following the manufacturer’s protocol with the following changes: a higher number of cells (10^7^) were harvested in combination with a decreased volume of Solubilization Buffer (0.2 mL) to achieve a higher concentration of membrane protein extracts.

### Western immunoblotting

SDS-PAGE and Western immunoblotting were carried out as previously described [74],with 50µg of cell membrane protein extract loaded per lane. Membranes were subjected to enhanced chemiluminescence (ECL prime, Cytiva #RPN2236) and developed using the ChemiDoc imaging system (Bio-Rad).

### Immunofluorescence (IF)

For IF, 10^5^ cells were crosslinked with 1% Paraformaldehyde (PFA) (Sigma-Aldrich, #P6148). Cells were next permeabilized and incubated with blocking 1% goat serum for 1 hour at room temperature and then with the primary antibodies overnight at 4°C. After four washes with PBS/0.05% Tween-20 for 15 minutes, the cells were incubated with the secondary antibodies diluted in the blocking buffer for 1 hour at 37°C. The cells were then washed four times with PBS for 15 minutes prior to cytospin and mounting with mounting medium containing 4′,6-diamidino-2-phenylindole (DAPI) (Abcam, #ab104139). Acquisition was made on an LSM700 Zeiss confocal microscope using Zen software at 60x magnification. Analysis was performed using Fiji.

### Antibodies used with Western blots and IF

The following antibodies were purchased from Santa Cruz Biotechnology (Santa Cruz, CA): N6 GATA1 (sc-265), M-20 FOG-1 (sc-9361; no longer available) and A-20 FOG-1 (sc-9362). The following antibodies were purchased from Proteintech: ABCG1 (13578-1-AP), ABCG5 (27722-1-AP), LDLR (10785-1-AP) and SREBP2 (28212-1-AP). The ATP1A1 antibody was purchased from Abcam (ab76020), the ABCA1 antibody from Novus Bio (NB400-105SS) and the NPM1 antibody was kindly provided by Pui K. Chan (Baylor College, Texas, USA). Secondary antibodies conjugated to horseradish peroxidase were purchased from DakoCytomation, Denmark (P0449 & P0162) and Santa Cruz Biotechnology (sc-2357 & sc-2314).

### Flow cytometry

Cells were analysed using a BD FACSCanto II or an LSR Fortessa flow cytometer (BD Biosciences) and acquired using the Diva Software version 9 (BD Biosciences). Data was analysed using the FlowJo software (v. 10.9.1, BD Biosciences). Erythroid differentiation was assessed by flow cytometry as described previously [75], with the only adaptation that CD44 extinction was used as a marker for diffrentiation, as MEL cells do not express Ter119*.* Representative examples of flow cytometric analysis are shown in S8 Fig.

### Cell cycle analysis

Cell cycle analysis was carried out by quantification of DNA content to estimate the percentage of proliferating versus G1 arrested terminally differentiated cells. Cells (10^6^) were fixed with 100% EtOH for 30 minutes at room temeprature. The fixed cells were stained with 500 μl of 50 μg/ml Propidium Iodide (PI; ThermoFisher, #BMS500PI) and 100 μg/ml RNase A (ThermoFisher, #EN0531) for 30 min at room temperature, prior to flow cytometry analysis.

### Apoptosis/Necrosis

Cells (5x10^5^) were centrifuged at 300g for 5 minutes and the supernatant was discarded. The cell pellet was then washed with 500 μl ice-cold 1x annexin buffer (Santa-Cruz, sc-291903). The cells were resuspended in 100 μl annexin buffer and 1 μl of Annexin V FITC (Santa-Cruz, sc-4252) with 1 μl of PI (ThermoFisher, #BMS500PI). This suspension was left to incubate at room temperature for 15 minutes in the dark and then 400 μl of 1x annexin buffer were added prior to flow cytometry analysis.

### Reactive Oxygen Species (ROS), Nile Red, Nile Red 12S (NR12S) and Membrane Fluidity assays

The following kits were used: for ROS, Abcam ab186029; for Nile Red, Abcam ab228553; for NR12S Cytoskeleton Inc. #MG08; for Membrane Fluidity, Abcam ab189819. For all assays, the manufacturers’ protocol was followed using 10^6^ cells, per assay. Analysis was carried out by flow cytometry, as above.

### Statistical analysis

Statistical analyses were performed with GraphPad Prism (version 7). The data was analyzed using the unpaired Mann-Whitney test.

## Supporting information

S1 File Counts matrix of differentially expressed genes in the FOG-1 knockout cells and list of genes for each subcluster shown in Fig 3. (XLSX)

S1 Table Numerical values supporting each graph. (XLSX)

S1 Fig Analysis of gene edits in the coding sequences of *Zfpm1* in the FOG-1 KO MEL cell clones G9, C8 and D3. **(A)** PCR analysis of genomic DNA of the first 5 exons of the *Zfpm1* gene edited CRISPR/Cas9. Expected amplified exon sizes are shown above the gel. **(B)** Example of an alignment of all *Zfpm1* exon 4 sequences extracted from the RNAseq data for each of the FOG-1 D3, C8, G9 KO MEL clones. Sequences are aligned to a reference sequence (WT exon), which is depicted as a dotted black line. Each nucleotide in the exon 4 sequence was assigned a colour, with white gaps representing missing sequence, presumably due to deletions. This analysis illustrates that clones D3 and C8 have a near complete deletion of exon 4, whereas the G9 clone has a partial deletion of this exon. **(C)** Top: Schematic representation of the WT FOG-1 protein sequence. Zinc finger domains 1 to 9 are indicated as dark blue boxes. Exons 1-6 coding for the N-terminal domain of FOG-1 are shown below the protein schematic in dark orange colour. Lower: schematic representation of the *Zfpm1* gene showing the exons that were affected by gene editing in the D3, G9 and C8 MEL FOG-1 KO clones, as deduced from the analysis of RNAseq data. A single diagonal line through an exon indicates a partial loss of sequence, a double diagonal line indicates a complete deletion of an exon. Asterisks (*) indicate a newly generated stop codon.
(TIF)

S2 Fig examples of expression profiles of specific genes in non-induced and DMSO-induced wild type MEL cells and in the FOG-1 KO clones D3 and C8. Blue dots correspond to WT profiles, green dots to FOG-1 KO clone D3 and orange dots to FOG-1 KO clone C8. G9 is not included in this analyses for reasons discussed in the main text.
(TIF)

S3 Fig Transcription factor (TF) target gene enrichment (ChEA) analysis. **(A)** ChEA analysis of A group genes shows an enrichment of gene targets for the erythroid TFs TAL-1 and GATA1. **(B)** ChEA analysis of B group genes shows an enrichment of gene targets for the megakaryocytic and myeloid TFs FLI1, RUNX1 and PU.1.
(TIF)

S4 Fig KEGG pathway analysis of genes in clusters A1 (panel A), A2 (panel B), A3 (panel C) and B (panel D) of the hierarchical clustering analysis in Fig 3. Up to top 10 pathways are shown in each case.
(TIF)

S5 Fig Heat map of expression proflies of GSEA gene sets for Heme metabolism (left panel), Cholesterol homeostasis (middle panel) and Myc targets (right panel). (TIF)

S6 Fig GATA1, FOG-1, K3K4me3, H3K4me1 and RNAPolII occupancies by ChIPseq of the *Abca1, Abcg1, Abcg5/8, Abca5, Ldlr* and *Srebf2* genes in DMSO-induced MEL cells. (PDF)

S7 Fig Western Blot analysis of HMGCS1 and HMGCR proteins in whole cell protein extracts from non-induced and DMSO-induced WT and FOG-1 KO MEL cell clones D3, C8 and G9. Actin was used as protein loading control.
(TIF)

S8 Fig (A) Representative flow cytometry plots for the data shown in Fig 1C. Non-induced WT and FOG-1 KO MEL cell clones D3, C8 and G9 are plotted based on cell size and CD44 staining intensity and are depicted in light blue colour. DMSO-induced WT and FOG-1 KO MEL cell clones D3, C8 and G9 are also plotted based on cell size and CD44 staining intensity and are depicted in red colour. **(B)** Representative flow cytometry plots for the data shown in Fig 1D. Non-induced and DMSO-induced WT and FOG-1 KO MEL cell clones D3, C8 and G9 were plotted based on propidium iodine staining intensity. **(C)** Representative flow cytometry plots for the data shown in Fig 1E. Non-induced and DMSO-induced WT and FOG-1 KO MEL cell clones D3, C8 and G9 were plotted based on propidium iodine and annexin staining intensity.
(TIF)

## References

[1] Chlon TM, Crispino JD. Combinatorial regulation of tissue specification by GATA and FOG factors. Development. 2012;139(21):3905–16. doi: 10.1242/dev.080440 23048181 PMC3472596

[2] Tsang AP, Visvader JE, Turner CA, Fujiwara Y, Yu C, Weiss MJ, et al. FOG, a multitype zinc finger protein, acts as a cofactor for transcription factor GATA-1 in erythroid and megakaryocytic differentiation. Cell. 1997;90(1):109–19. doi: 10.1016/s0092-8674(00)80318-9 9230307

[3] Tsang AP, Fujiwara Y, Hom DB, Orkin SH. Failure of megakaryopoiesis and arrested erythropoiesis in mice lacking the GATA-1 transcriptional cofactor FOG. Genes Dev. 1998;12(8):1176–88. doi: 10.1101/gad.12.8.1176 9553047 PMC316724

[4] Rodriguez P, Bonte E, Krijgsveld J, Kolodziej KE, Guyot B, Heck AJR, et al. GATA-1 forms distinct activating and repressive complexes in erythroid cells. EMBO J. 2005;24(13):2354–66. doi: 10.1038/sj.emboj.7600702 15920471 PMC1173143

[5] Hong W, Nakazawa M, Chen Y-Y, Kori R, Vakoc CR, Rakowski C, et al. FOG-1 recruits the NuRD repressor complex to mediate transcriptional repression by GATA-1. EMBO J. 2005;24(13):2367–78. doi: 10.1038/sj.emboj.7600703 15920470 PMC1173144

[6] Katz SG, Cantor AB, Orkin SH. Interaction between FOG-1 and the corepressor C-terminal binding protein is dispensable for normal erythropoiesis in vivo. Mol Cell Biol. 2002;22(9):3121–8. doi: 10.1128/MCB.22.9.3121-3128.2002 11940669 PMC133767

[7] Clifton MK, Westman BJ, Thong SY, O’Connell MR, Webster MW, Shepherd NE, et al. The identification and structure of an N-terminal PR domain show that FOG1 is a member of the PRDM family of proteins. PLoS One. 2014;9(8):e106011. doi: 10.1371/journal.pone.0106011 25162672 PMC4146578

[8] Snow J, Orkin S. Translational isoforms of FOG1 regulate GATA1-interacting complexes. J Biol Chem. 2009;284(43):29310–9. doi: 10.1074/jbc.M109.043497 PMID: 19654328 PMID: 19654328 PMC2785561

[9] Pevny L, Lin CS, D’Agati V, Simon MC, Orkin SH, Costantini F. Development of hematopoietic cells lacking transcription factor GATA-1. Development. 1995;121(1):163–72. doi: 10.1242/dev.121.1.163 7867497

[10] Pevny L, Simon MC, Robertson E, Klein WH, Tsai SF, D’Agati V, et al. Erythroid differentiation in chimaeric mice blocked by a targeted mutation in the gene for transcription factor GATA-1. Nature. 1991;349(6306):257–60. doi: 10.1038/349257a0 1987478

[11] Vyas P, Ault K, Jackson CW, Orkin SH, Shivdasani RA. Consequences of GATA-1 Deficiency in Megakaryocytes and Platelets. Blood. 1999;93(9):2867–75. doi: 10.1182/blood.v93.9.2867 PMID: 10216081

[12] Shivdasani RA, Fujiwara Y, McDevitt MA, Orkin SH. A lineage-selective knockout establishes the critical role of transcription factor GATA-1 in megakaryocyte growth and platelet development. EMBO J. 1997;16(13):3965–73. doi: 10.1093/emboj/16.13.3965 9233806 PMC1170020

[13] Crispino JD, Lodish MB, MacKay JP, Orkin SH. Use of altered specificity mutants to probe a specific protein-protein interaction in differentiation: the GATA-1:FOG complex. Mol Cell. 1999;3(2):219–28. doi: 10.1016/s1097-2765(00)80312-3 10078204

[14] Chang AN, Cantor AB, Fujiwara Y, Lodish MB, Droho S, Crispino JD, et al. GATA-factor dependence of the multitype zinc-finger protein FOG-1 for its essential role in megakaryopoiesis. Proc Natl Acad Sci U S A. 2002;99(14):9237–42. doi: 10.1073/pnas.142302099 PMID: 12077323 PMC123124

[15] Nichols KE, Crispino JD, Poncz M, White JG, Orkin SH, Maris JM, et al. Familial dyserythropoietic anaemia and thrombocytopenia due to an inherited mutation in GATA1. Nat Genet. 2000;24(3):266–70. doi: 10.1038/73480 10700180 PMC10576470

[16] Pal S, Cantor AB, Johnson KD, Moran TB, Boyer ME, Orkin SH, et al. Coregulator-dependent facilitation of chromatin occupancy by GATA-1. Proc Natl Acad Sci U S A. 2004;101(4):980–5. doi: 10.1073/pnas.0307612100 14715908 PMC327128

[17] Letting DL, Chen Y-Y, Rakowski C, Reedy S, Blobel GA. Context-dependent regulation of GATA-1 by friend of GATA-1. Proc Natl Acad Sci U S A. 2004;101(2):476–81. doi: 10.1073/pnas.0306315101 14695898 PMC327172

[18] Chlon TM, Doré LC, Crispino JD. Cofactor-mediated restriction of GATA-1 chromatin occupancy coordinates lineage-specific gene expression. Mol Cell. 2012;47(4):608–21. doi: 10.1016/j.molcel.2012.05.051 22771118 PMC3432917

[19] Vakoc CR, Letting DL, Gheldof N, Sawado T, Bender MA, Groudine M, et al. Proximity among distant regulatory elements at the beta-globin locus requires GATA-1 and FOG-1. Mol Cell. 2005;17(3):453–62. doi: 10.1016/j.molcel.2004.12.028 PMID: 15694345

[20] Jing H, Vakoc CR, Ying L, Mandat S, Wang H, Zheng X, et al. Exchange of GATA factors mediates transitions in looped chromatin organization at a developmentally regulated gene locus. Mol Cell. 2008;29(2):232–42. doi: 10.1016/j.molcel.2007.11.020 18243117 PMC2254447

[21] Miccio A, Wang Y, Hong W, Gregory GD, Wang H, Yu X, et al. NuRD mediates activating and repressive functions of GATA-1 and FOG-1 during blood development. EMBO J. 2010;29(2):442–56. doi: 10.1038/emboj.2009.336 19927129 PMC2824460

[22] Garriga-Canut M, Orkin SH. Transforming acidic coiled-coil protein 3 (TACC3) controls friend of GATA-1 (FOG-1) subcellular localization and regulates the association between GATA-1 and FOG-1 during hematopoiesis. J Biol Chem. 2004;279(22):23597–605. doi: 10.1074/jbc.M313987200 15037632

[23] Chen K, Liu J, Heck S, Chasis J, An X, Mohandas N. Resolving the distinct stages in erythroid differentiation based on dynamic changes in membrane protein expression during erythropoiesis. Proc Natl Acad Sci U S A. 2009;106(41):17413–8. doi: 10.1073/pnas.0906886106 PMID: 19805084 PMC2762680

[24] Gregory T, Yu C, Ma A, Orkin SH, Blobel GA, Weiss MJ. GATA-1 and erythropoietin cooperate to promote erythroid cell survival by regulating bcl-xL expression. Blood. 1999;94(1):87–96. doi: 10.1182/blood.v94.1.87.413k41_87_96 10381501

[25] Seo MJ, Liu X, Chang M, Park JH. GATA-binding protein 1 is a novel transcription regulator of peroxiredoxin 5 in human breast cancer cells. Int J Oncol. 2012;40(3):655–64. doi: 10.3892/ijo.2011.1236 22020876

[26] Ge SX, Jung D, Yao R. ShinyGO: a graphical gene-set enrichment tool for animals and plants. Bioinformatics. 2020;36(8):2628–9. doi: 10.1093/bioinformatics/btz931 31882993 PMC7178415

[27] Lachmann A, Xu H, Krishnan J, Berger SI, Mazloom AR, Ma’ayan A. ChEA: transcription factor regulation inferred from integrating genome-wide ChIP-X experiments. Bioinformatics. 2010;26(19):2438–44. doi: 10.1093/bioinformatics/btq466 20709693 PMC2944209

[28] Subramanian A, Tamayo P, Mootha V, Mukherjee S, Ebert B, Gillette M, et al. Gene set enrichment analysis: a knowledge-based approach for interpreting genome-wide expression profiles. Proc Natl Acad Sci U S A. 2005;102(43):15545–50. 10.1073/pnas.0506580102 16199517 PMC1239896

[29] Mootha VK, Lindgren CM, Eriksson K-F, Subramanian A, Sihag S, Lehar J, et al. PGC-1alpha-responsive genes involved in oxidative phosphorylation are coordinately downregulated in human diabetes. Nat Genet. 2003;34(3):267–73. doi: 10.1038/ng1180 12808457

[30] Rylski M, Welch JJ, Chen Y-Y, Letting DL, Diehl JA, Chodosh LA, et al. GATA-1-mediated proliferation arrest during erythroid maturation. Mol Cell Biol. 2003;23(14):5031–42. doi: 10.1128/MCB.23.14.5031-5042.2003 12832487 PMC162202

[31] Xu L, Wu F, Yang L, Wang F, Zhang T, Deng X. miR-144/451 inhibits c-Myc to promote erythroid differentiation. FASEB Journal. 2020;34(10):13194–210. doi: 10.1096/fj.202000941R PMID: 33319407

[32] Shirihai OS, Gregory T, Yu C, Orkin SH, Weiss MJ. ABC-me: a novel mitochondrial transporter induced by GATA-1 during erythroid differentiation. EMBO J. 2000;19(11):2492–502. doi: 10.1093/emboj/19.11.2492 10835348 PMC212759

[33] Yamamoto M, Arimura H, Fukushige T, Minami K, Nishizawa Y, Tanimoto A. Abcb10 role in heme biosynthesis in vivo: Abcb10 knockout in mice causes anemia with protoporphyrin IX and iron accumulation. Mol Cell Biol. 2014;34(6):1077–84. doi: 10.1128/MCB.01345-13 PMID: 24421385 PMC3958026

[34] Krishnamurthy PC, Du G, Fukuda Y, Sun D, Sampath J, Mercer KE, et al. Identification of a mammalian mitochondrial porphyrin transporter. Nature. 2006;443(7111):586–9. doi: 10.1038/nature05125 17006453

[35] Zhao C, Haase W, Tampe R, Abele R. Peptide specificity and lipid activation of the lysosomal transport complex ABCB9 (TAPL). J Biol Chem. 2008;283(25):17083–91. doi: 10.1074/jbc.M801794200 18434309

[36] Wang N, Westerterp M. ABC Transporters, Cholesterol Efflux, and Implications for Cardiovascular Diseases. Adv Exp Med Biol. 2020;1276:67–83. doi: 10.1007/978-981-15-6082-8_6 32705595

[37] Yu M, Riva L, Xie H, Schindler Y, Moran TB, Cheng Y, et al. Insights into GATA-1-mediated gene activation versus repression via genome-wide chromatin occupancy analysis. Mol Cell. 2009;36(4):682–95. doi: 10.1016/j.molcel.2009.11.002 19941827 PMC2800995

[38] Tamehiro N, Shigemoto-Mogami Y, Kakeya T, Okuhira K-I, Suzuki K, Sato R, et al. Sterol regulatory element-binding protein-2- and liver X receptor-driven dual promoter regulation of hepatic ABC transporter A1 gene expression: mechanism underlying the unique response to cellular cholesterol status. J Biol Chem. 2007;282(29):21090–9. doi: 10.1074/jbc.M701228200 17526932

[39] Smith JR, Osborne TF, Goldstein JL, Brown MS. Identification of nucleotides responsible for enhancer activity of sterol regulatory element in low density lipoprotein receptor gene. J Biol Chem. 1990;265(4):2306–10. doi: 10.1016/s0021-9258(19)39976-4 2298751

[40] Weber L-W, Boll M, Stampfl A. Maintaining cholesterol homeostasis: sterol regulatory element-binding proteins. World J Gastroenterol. 2004;10(21):3081–7. doi: 10.3748/wjg.v10.i21.3081 15457548 PMC4611246

[41] Lu Z, Huang L, Li Y, Xu Y, Zhang R, Zhou Q, et al. Fine-Tuning of Cholesterol Homeostasis Controls Erythroid Differentiation. Adv Sci (Weinh). 2022;9(2):e2102669. doi: 10.1002/advs.202102669 34739188 PMC8805577

[42] Yajima Y, Sato M, Sorimachi H, Inomata M, Maki M, Kawashima S. Calpain system regulates the differentiation of adult primitive mesenchymal ST-13 adipocytes. Endocrinology. 2006;147(10):4811–9. doi: 10.1210/en.2005-1647 16857754

[43] Rittmann LS, Jelsema CL, Schwartz EL, Tsiftsoglou AS, Sartorelli AC. Lipid composition of Friend leukemia cells following induction of erythroid differentiation by dimethyl sulfoxide. J Cell Physiol. 1982;110(1):50–5. doi: 10.1002/jcp.1041100109 7068766

[44] Weiss M, Yu C, Orkin S. Erythroid-cell-specific properties of transcription factor GATA-1 revealed by phenotypic rescue of a gene-targeted cell line. Mol Cell Biol. 1997;17(3):1642–51. doi: 10.1128/MCB.17.3.1642 9032291 PMC231889

[45] Tshori S, Nechushtan H. Mast cell transcription factors--regulators of cell fate and phenotype. Biochim Biophys Acta. 2012;1822(1):42–8. doi: 10.1016/j.bbadis.2010.12.024 21236338

[46] Cantor AB, Iwasaki H, Arinobu Y, Moran TB, Shigematsu H, Sullivan MR, et al. Antagonism of FOG-1 and GATA factors in fate choice for the mast cell lineage. J Exp Med. 2008;205(3):611–24. doi: 10.1084/jem.20070544 18299398 PMC2275384

[47] Sugiyama D, Tanaka M, Kitajima K, Zheng J, Yen H, Murotani T, et al. Differential context-dependent effects of friend of GATA-1 (FOG-1) on mast-cell development and differentiation. Blood. 2008;111(4):1924–32. doi: 10.1182/blood-2007-08-104489 18063754

[48] Querfurth E, Schuster M, Kulessa H, Crispino JD, Döderlein G, Orkin SH, et al. Antagonism between C/EBPbeta and FOG in eosinophil lineage commitment of multipotent hematopoietic progenitors. Genes Dev. 2000;14(19):2515–25. doi: 10.1101/gad.177200 11018018 PMC316981

[49] Cooper RA. Influence of increased membrane cholesterol on membrane fluidity and cell function in human red blood cells. J Supramol Struct. 1978;8(4):413–30. doi: 10.1002/jss.400080404 723275

[50] Luo J, Yang H, Song BL. Mechanisms and regulation of cholesterol homeostasis. Nat Rev Mol Cell Biol. 2020;21(4):225–45. doi: 10.1038/s41580-019-0190-7 PMID: 31848472

[51] Russell DW. Fifty years of advances in bile acid synthesis and metabolism. J Lipid Res. 2009;50 Suppl(Suppl):S120-5. doi: 10.1194/jlr.R800026-JLR200 18815433 PMC2674696

[52] Westerterp M, Gourion-Arsiquaud S, Murphy AJ, Shih A, Cremers S, Levine RL, et al. Regulation of hematopoietic stem and progenitor cell mobilization by cholesterol efflux pathways. Cell Stem Cell. 2012;11(2):195–206. doi: 10.1016/j.stem.2012.04.024 22862945 PMC3413200

[53] Tall AR, Yvan-Charvet L, Westerterp M, Murphy AJ. Cholesterol efflux: a novel regulator of myelopoiesis and atherogenesis. Arterioscler Thromb Vasc Biol. 2012;32(11):2547–52. doi: 10.1161/ATVBAHA.112.300134 23077140

[54] Potter JE, James MJ, Kandutsch AA. Sequential cycles of cholesterol and dolichol synthesis in mouse spleens during phenylhydrazine-induced erythropoiesis. J Biol Chem. 1981;256(5):2371–6. doi: 10.1016/s0021-9258(19)69789-9 7462243

[55] Quintana AM, Picchione F, Klein Geltink RI, Taylor MR, Grosveld GC. Zebrafish ETV7 regulates red blood cell development through the cholesterol synthesis pathway. Dis Models Mech. 2014;7(2):265–70. doi: 10.1242/dmm.012526 PMID: 24357328 PMC3917247

[56] Hernandez JA, Castro VL, Reyes-Nava N, Montes LP, Quintana AM. Mutations in the zebrafish hmgcs1 gene reveal a novel function for isoprenoids during red blood cell development. Blood Adv. 2019;3(8):1244–54. doi: 10.1182/bloodadvances.2018024539 30987969 PMC6482358

[57] Kosters A, Kunne C, Looije N, Patel SB, Oude Elferink RPJ, Groen AK. The mechanism of ABCG5/ABCG8 in biliary cholesterol secretion in mice. J Lipid Res. 2006;47(9):1959–66. doi: 10.1194/jlr.M500511-JLR200 16741293 PMC1805467

[58] Hung KT, Berisha SZ, Ritchey BM, Santore J, Smith JD. Red blood cells play a role in reverse cholesterol transport. Arterioscler Thromb Vasc Biol. 2012;32(6):1460–5. doi: 10.1161/ATVBAHA.112.248971 22499994 PMC3360517

[59] Lai S-J, Ohkawa R, Horiuchi Y, Kubota T, Tozuka M. Red blood cells participate in reverse cholesterol transport by mediating cholesterol efflux of high-density lipoprotein and apolipoprotein A-I from THP-1 macrophages. Biol Chem. 2019;400(12):1593–602. doi: 10.1515/hsz-2019-0244 31188743

[60] Morgan PK, Fang L, Lancaster GI, Murphy AJ. Hematopoiesis is regulated by cholesterol efflux pathways and lipid rafts: connections with cardiovascular diseases. J Lipid Res. 2020;61(5):667–75. doi: 10.1194/jlr.TR119000267 31471447 PMC7193969

[61] Tsiftsoglou A, Housman D, Wong W. The inhibition of commitment of mouse erythroleukemia cells by steroids involves a glucocorticoid-receptor mediated process(es) acting at the nuclear level. Biochim Biophys Acta. 1986;889(2):251–61. doi: 10.1016/0167-4889(86)90111-4 3465373

[62] Mejia-Pous C, Damiola F, Gandrillon O. Cholesterol synthesis-related enzyme oxidosqualene cyclase is required to maintain self-renewal in primary erythroid progenitors. Cell Prolif. 2011;44(5):441–52. doi: 10.1111/j.1365-2184.2011.00771.x 21951287 PMC6495882

[63] Ohkawa R, Low H, Mukhamedova N, Fu Y, Lai S-J, Sasaoka M, et al. Cholesterol transport between red blood cells and lipoproteins contributes to cholesterol metabolism in blood. J Lipid Res. 2020;61(12):1577–88. doi: 10.1194/jlr.RA120000635 32907987 PMC7707172

[64] Yamauchi Y, Iwamoto N, Rogers M, Abe-Dohmae S, Fujimoto T, Chang C. Deficiency in the lipid exporter ABCA1 impairs retrograde sterol movement and disrupts sterol sensing at the endoplasmic reticulum. J Biol Chem. 2015;290(39):23464–77. doi: 10.1074/jbc.M115.662668 PMID: 26198636 PMC4583027

[65] Antoniou M. Induction of Erythroid-Specific Expression in Murine Erythroleukemia (MEL) Cell Lines. Methods Mol Biol. 1991;7 421–34. doi: 10.1385/0-89603-178-0:421 21416373

[66] Welch JJ, Watts JA, Vakoc CR, Yao Y, Wang H, Hardison RC, et al. Global regulation of erythroid gene expression by transcription factor GATA-1. Blood. 2004;104(10):3136–47. doi: 10.1182/blood-2004-04-1603 15297311

[67] Ran FA, Hsu PD, Wright J, Agarwala V, Scott DA, Zhang F. Genome engineering using the CRISPR-Cas9 system. Nat Protoc. 2013;8(11):2281–308. doi: 10.1038/nprot.2013.143 24157548 PMC3969860

[68] Bolger AM, Lohse M, Usadel B. Trimmomatic: a flexible trimmer for Illumina sequence data. Bioinformatics. 2014;30(15):2114–20. doi: 10.1093/bioinformatics/btu170 24695404 PMC4103590

[69] Patro R, Duggal G, Love MI, Irizarry RA, Kingsford C. Salmon provides fast and bias-aware quantification of transcript expression. Nat Methods. 2017;14(4):417–9. doi: 10.1038/nmeth.4197 28263959 PMC5600148

[70] Love MI, Soneson C, Hickey PF, Johnson LK, Pierce NT, Shepherd L, et al. Tximeta: Reference sequence checksums for provenance identification in RNA-seq. PLoS Comput Biol. 2020;16(2):e1007664. doi: 10.1371/journal.pcbi.1007664 32097405 PMC7059966

[71] Kucukural A, Yukselen O, Ozata DM, Moore MJ, Garber M. DEBrowser: interactive differential expression analysis and visualization tool for count data. BMC Genomics. 2019;20(1):6. doi: 10.1186/s12864-018-5362-x 30611200 PMC6321710

[72] Yu G, Wang L-G, Han Y, He Q-Y. clusterProfiler: an R package for comparing biological themes among gene clusters. OMICS. 2012;16(5):284–7. doi: 10.1089/omi.2011.0118 22455463 PMC3339379

[73] Lavery DJ, Schibler U. Circadian transcription of the cholesterol 7 alpha hydroxylase gene may involve the liver-enriched bZIP protein DBP. Genes Dev. 1993;7(10):1871–84. doi: 10.1101/gad.7.10.1871 8405996

[74] Rodriguez P, Braun H, Kolodziej KE, de Boer E, Campbell J, Bonte E, et al. Isolation of transcription factor complexes by in vivo biotinylation tagging and direct binding to streptavidin beads. Methods Mol Biol. 2006;338:305–23. doi: 10.1385/1-59745-097-9:305 16888367

[75] Liu J, Zhang J, Ginzburg Y, Li H, Xue F, De Franceschi L, et al. Quantitative analysis of murine terminal erythroid differentiation in vivo: novel method to study normal and disordered erythropoiesis. Blood. 2013;121(8):e43-9. doi: 10.1182/blood-2012-09-456079 23287863 PMC3578961

