# Supplementary figures and images for: A novel role for Friend of GATA1 (FOG-1) in regulating cholesterol transport in murine erythropoiesis
Source: PLoS Genet. 2025 Mar 6;21(3):e1011617. doi: 10.1371/journal.pgen.1011617 (PMC11913303; doi:10.1371/journal.pgen.1011617)

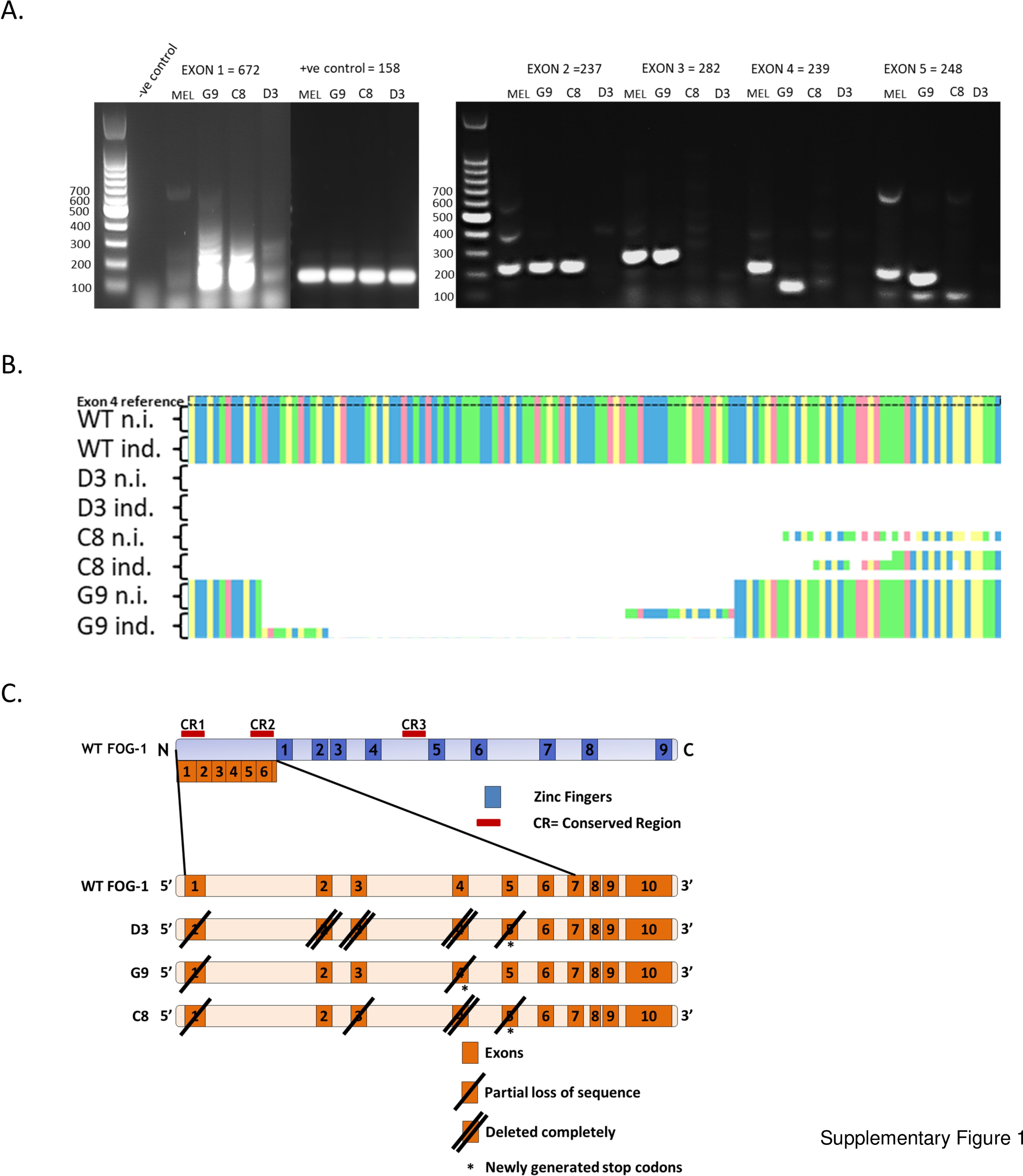

Supplement: S1 Fig — (A) PCR analysis of genomic DNA of the first 5 exons of the Zfpm1 gene edited CRISPR/Cas9. Expected amplified exon sizes are shown above the gel. (B) Example of an alignment of all Zfpm1 exon 4 sequences extracted from the RNAseq data for each of the FOG-1 D3, C8, G9 KO MEL clones. Sequences are aligned to a reference sequence (WT exon), which is depicted as a dotted black line. Each nucleotide in the exon 4 sequence was assigned a colour, with white gaps representing missing sequence, presumably due to deletions. This analysis illustrates that clones D3 and C8 have a near complete deletion of exon 4, whereas the G9 clone has a partial deletion of this exon. (C) Top: Schematic representation of the WT FOG-1 protein sequence. Zinc finger domains 1 to 9 are indicated as dark blue boxes. Exons 1-6 coding for the N-terminal domain of FOG-1 are shown below the protein schematic in dark orange colour. Lower: schematic representation of the Zfpm1 gene showing the exons that were affected by gene editing in the D3, G9 and C8 MEL FOG-1 KO clones, as deduced from the analysis of RNAseq data. A single diagonal line through an exon indicates a partial loss of sequence, a double diagonal line indicates a complete deletion of an exon. Asterisks (*) indicate a newly generated stop codon. (TIF) [file pgen.1011617.s003.tif]

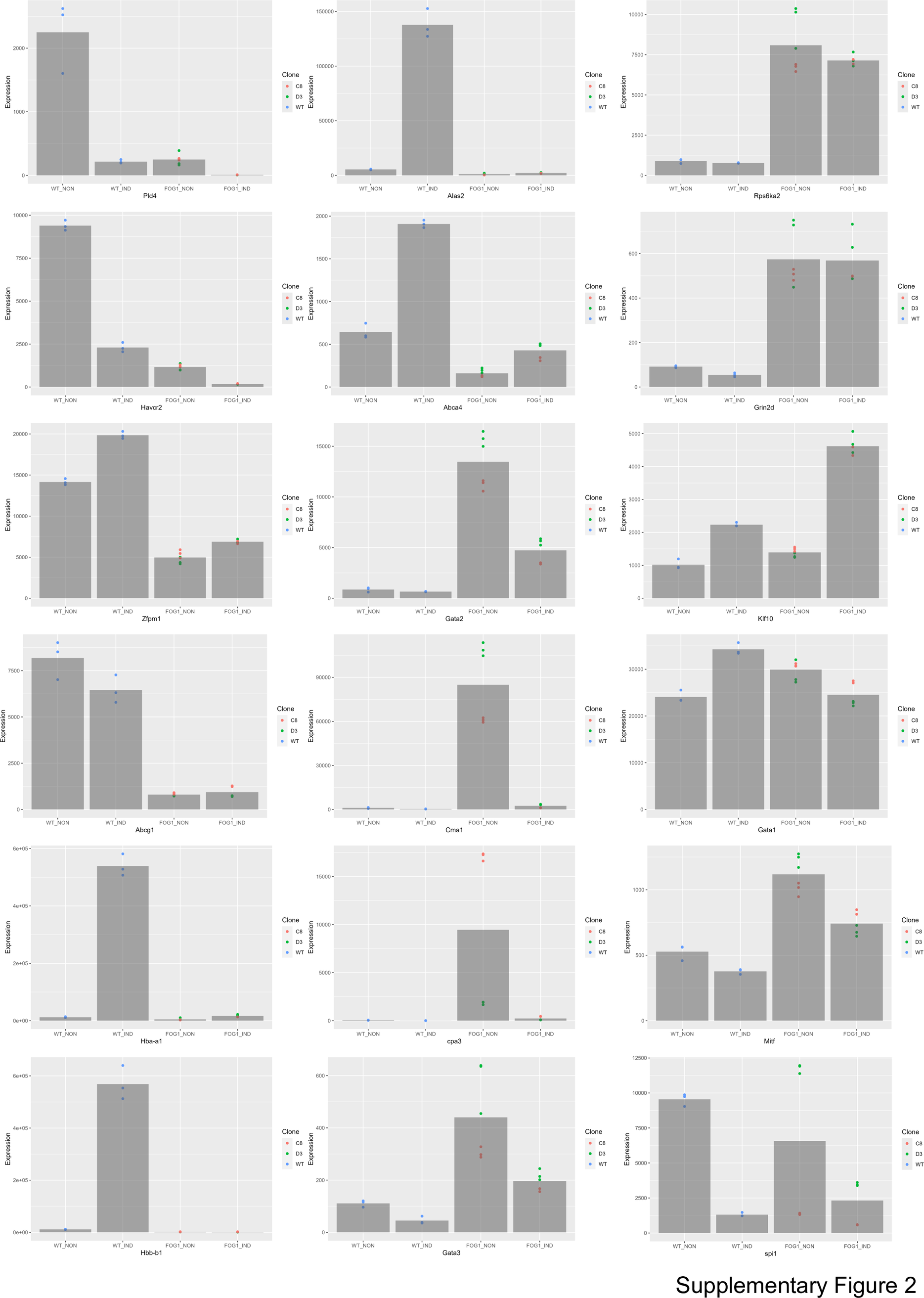

Supplement: S2 Fig — Blue dots correspond to WT profiles, green dots to FOG-1 KO clone D3 and orange dots to FOG-1 KO clone C8. G9 is not included in this analyses for reasons discussed in the main text. (TIF) [file pgen.1011617.s004.tif]

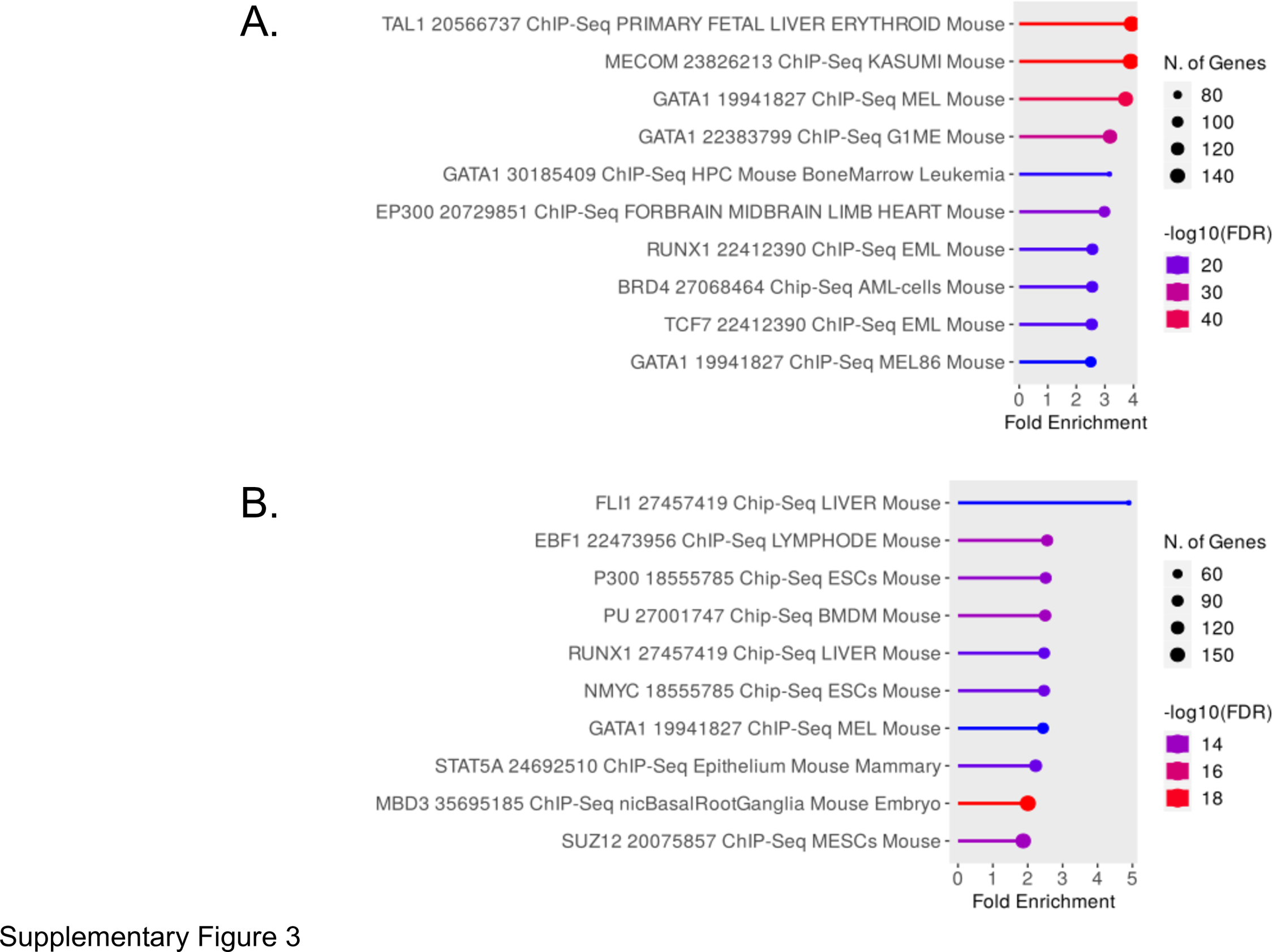

Supplement: S3 Fig — (A) ChEA analysis of A group genes shows an enrichment of gene targets for the erythroid TFs TAL-1 and GATA1. (B) ChEA analysis of B group genes shows an enrichment of gene targets for the megakaryocytic and myeloid TFs FLI1, RUNX1 and PU.1. (TIF) [file pgen.1011617.s005.tif]

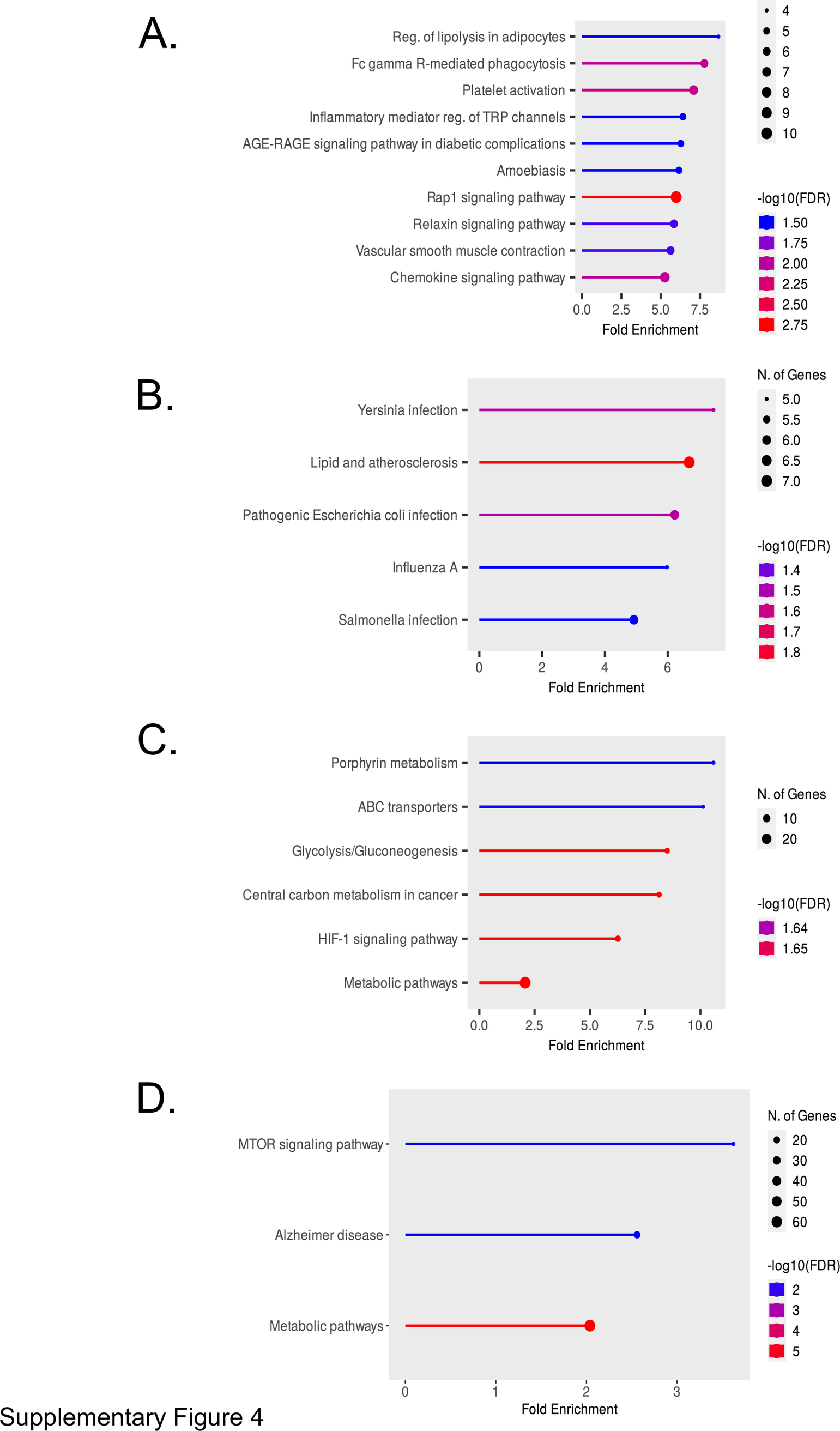

Supplement: S4 Fig — Up to top 10 pathways are shown in each case. (TIF) [file pgen.1011617.s006.tif]

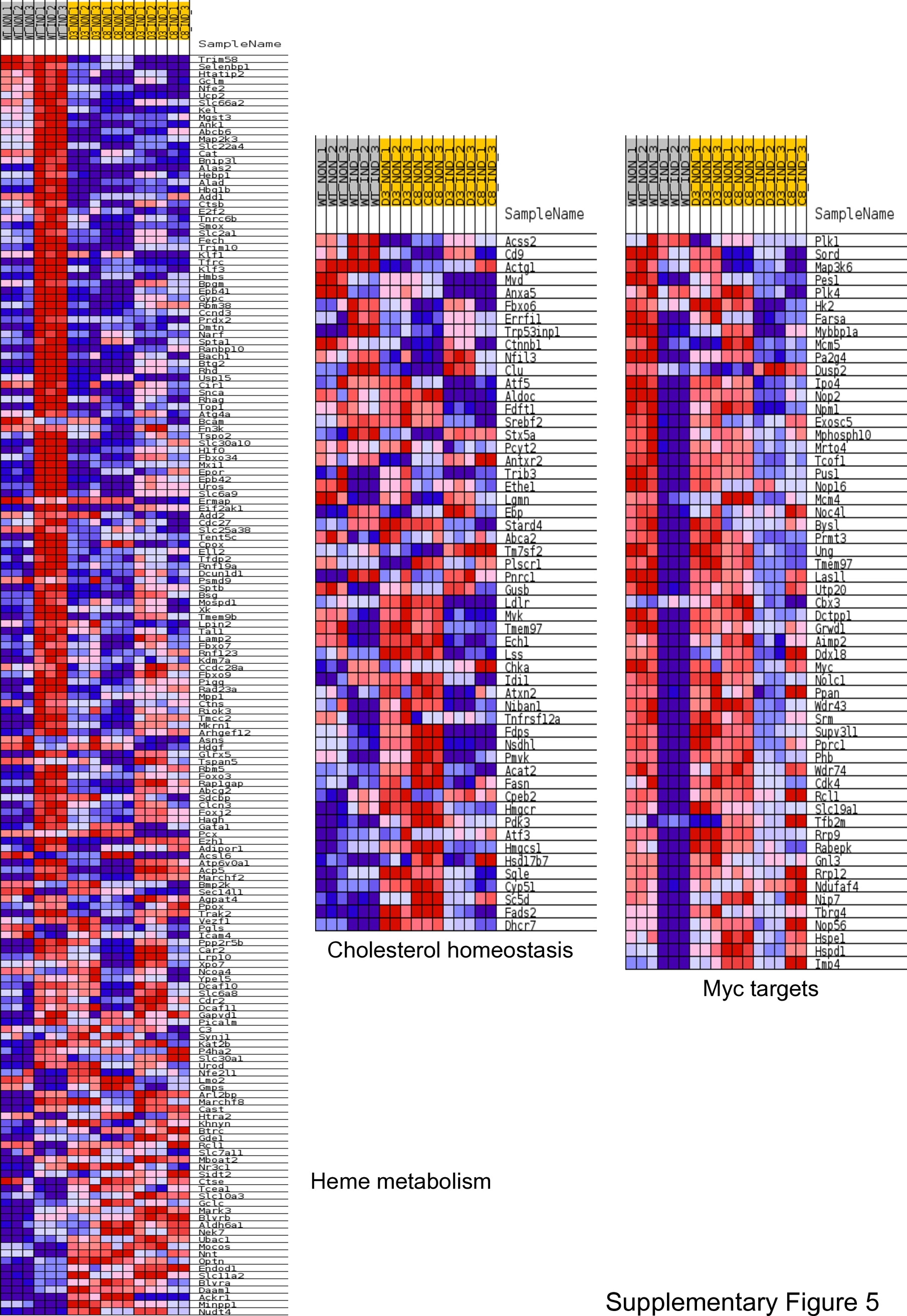

Supplement: S5 Fig — (TIF) [file pgen.1011617.s007.tif]

# Abca1

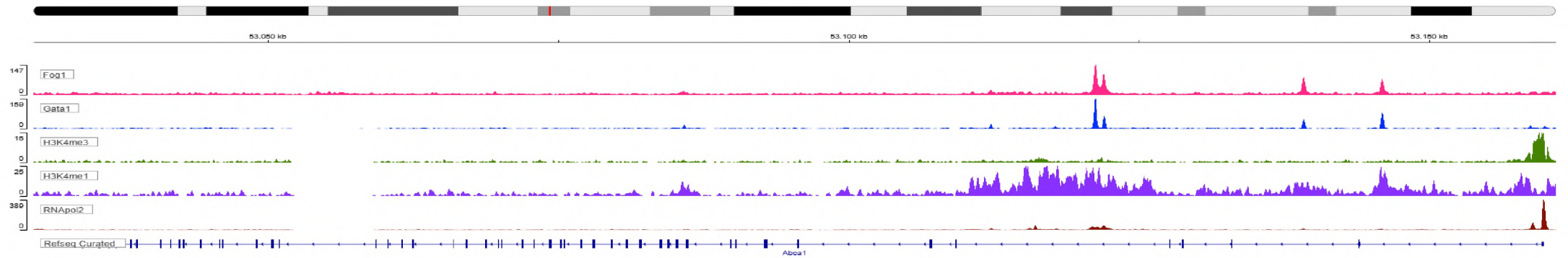

# Ldlr

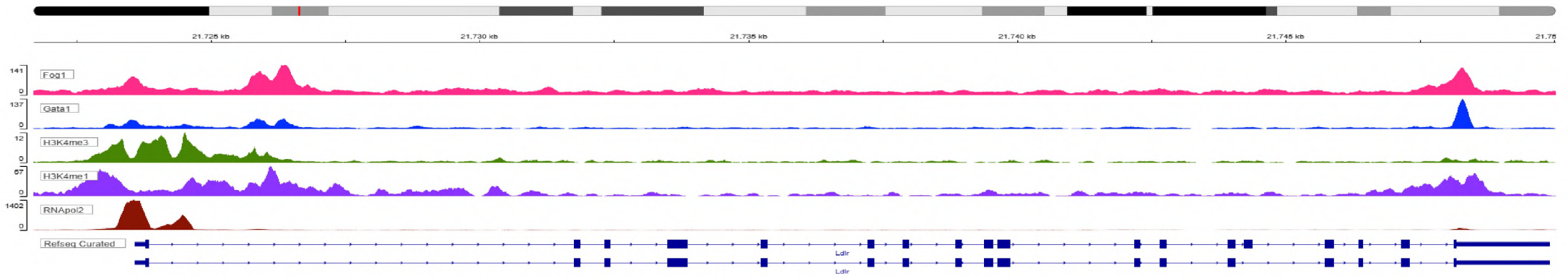

# Srebp2

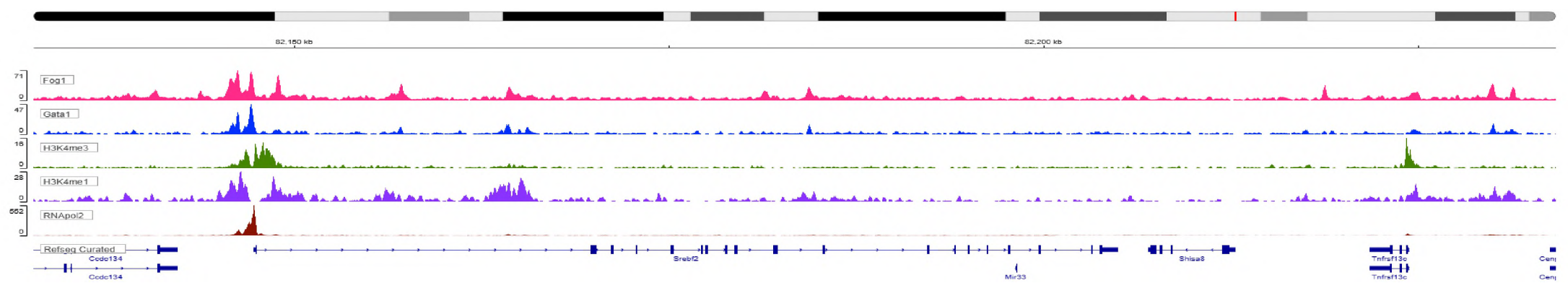

## Abca5

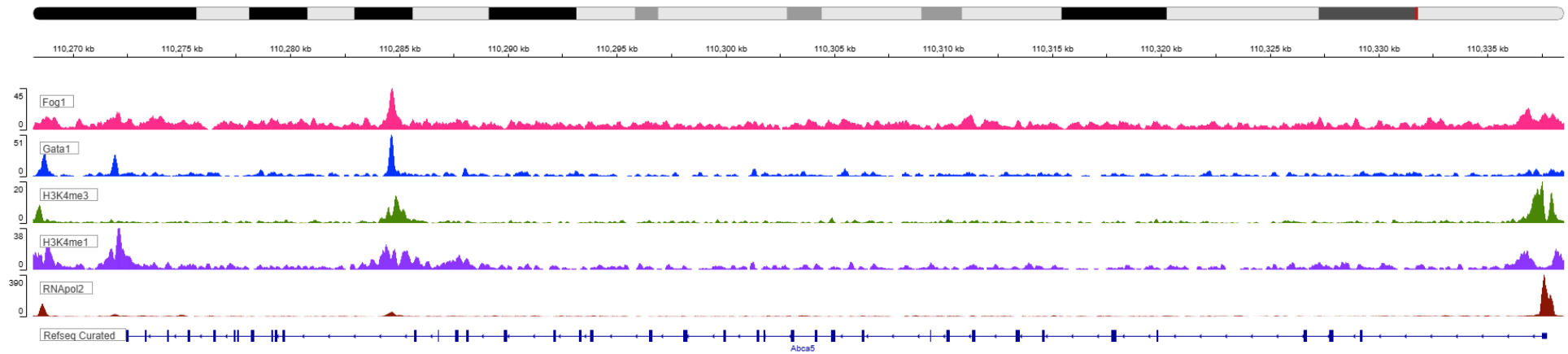

## Abcg1

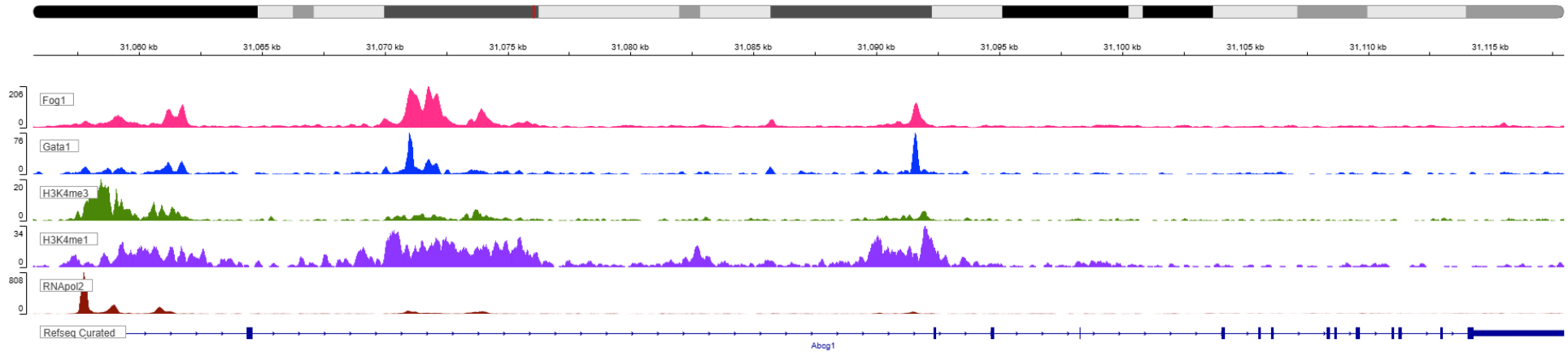

## Abcg5/Abcg8

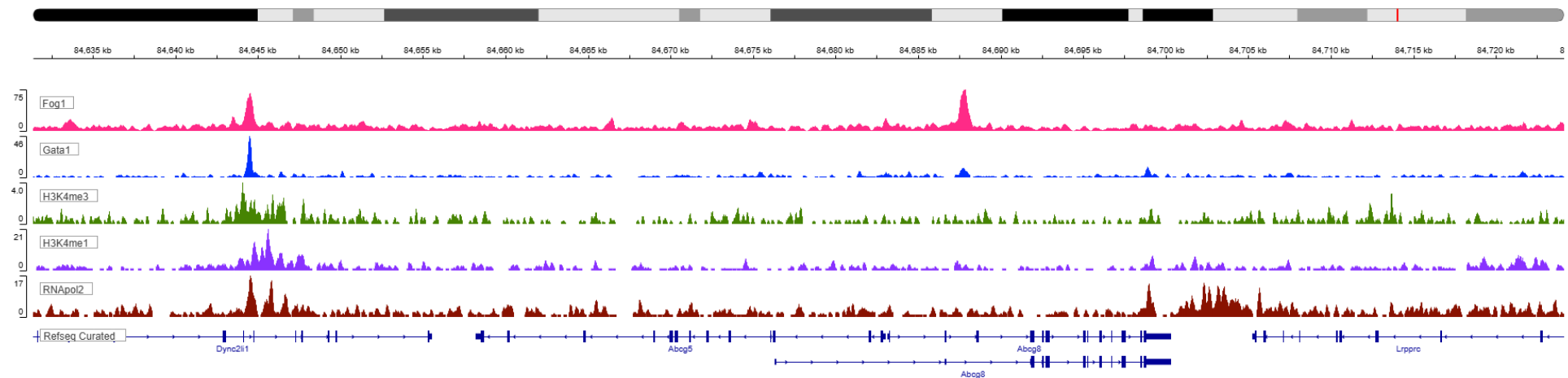

Supplement: S6 Fig — (PDF) [file pgen.1011617.s008.pdf]

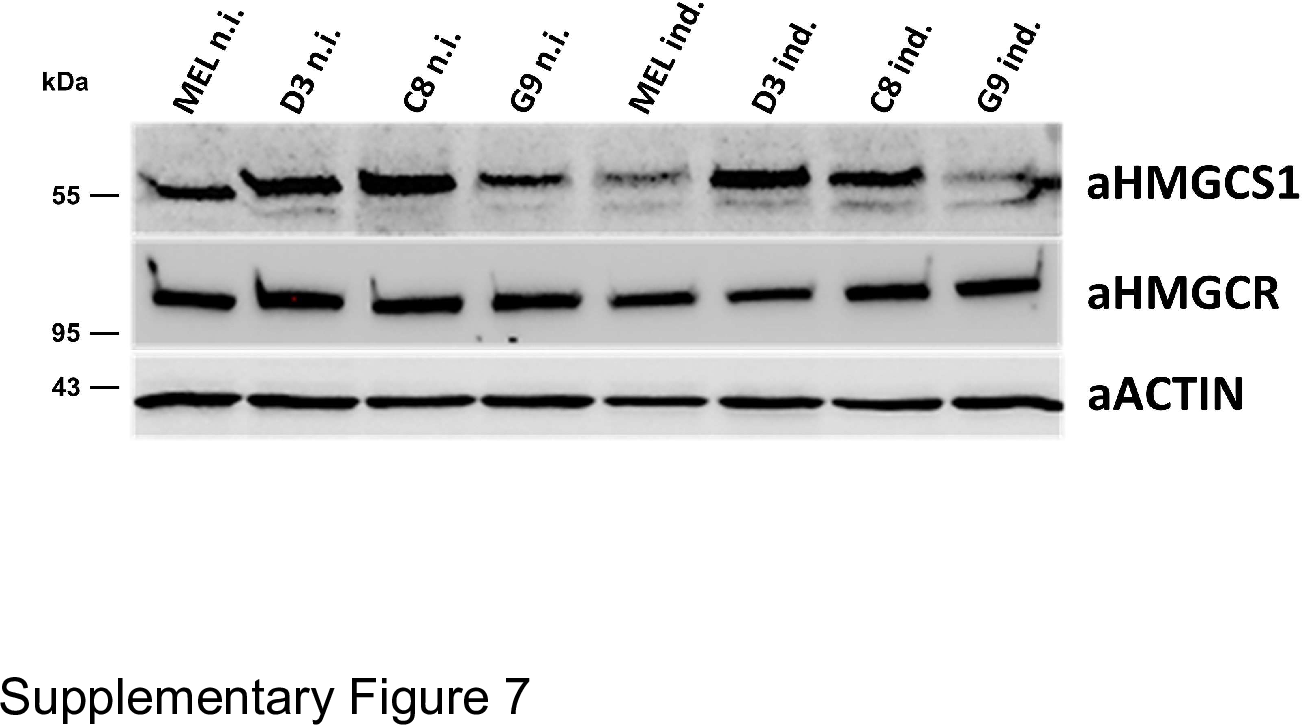

Supplement: S7 Fig — Actin was used as protein loading control. (TIF) [file pgen.1011617.s009.tif]

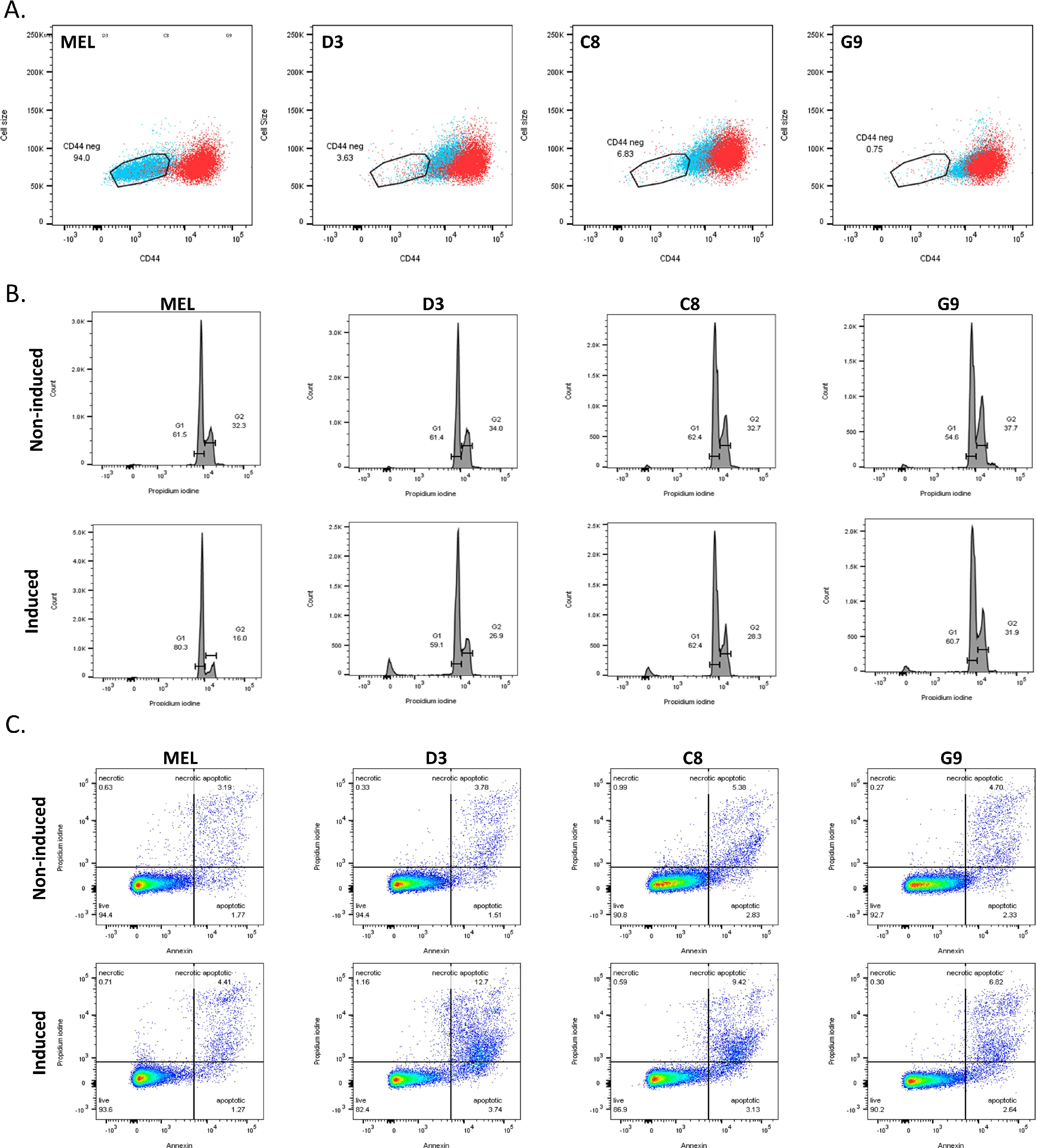

Supplement: S8 Fig — Non-induced WT and FOG-1 KO MEL cell clones D3, C8 and G9 are plotted based on cell size and CD44 staining intensity and are depicted in light blue colour. DMSO-induced WT and FOG-1 KO MEL cell clones D3, C8 and G9 are also plotted based on cell size and CD44 staining intensity and are depicted in red colour. (B) Representative flow cytometry plots for the data shown in Fig 1D. Non-induced and DMSO-induced WT and FOG-1 KO MEL cell clones D3, C8 and G9 were plotted based on propidium iodine staining intensity. (C) Representative flow cytometry plots for the data shown in Fig 1E. Non-induced and DMSO-induced WT and FOG-1 KO MEL cell clones D3, C8 and G9 were plotted based on propidium iodine and annexin staining intensity. (TIF) [file pgen.1011617.s010.tif]
